# Electret‐Inspired Charge‐Injected Hydrogel for Scar‐Free Healing of Bacterially Infected Burns Through Bioelectrical Stimulation and Immune Modulation

**DOI:** 10.1002/advs.202411889

**Published:** 2025-02-14

**Authors:** Mujie Liu, Yuheng Wang, Haodong Wang, Lihong Qi, Yuxuan Shang, Jiajie Song, Xiulong Feng, Yiwei Chen, Waqar Ali Memon, Yuping Shen, Xiaodong Wu, Jiangbei Cao, Yifan Zhao, Zhuangde Jiang, Dingxin Liu, Shareen Shafique, Shengtao Li, Guanghao Lu, Zhixiang Wei, Zhijie Liu, Kun Zhou, Yuping Quan, Xiaoyu Zhang, Xin Zou, Xuefeng Wang, Na Liu, Yaqing Zhang, Yiwei Hu, Chao Han, Wen Wang

**Affiliations:** ^1^ Functional and Molecular Imaging Key Lab of Shaanxi Province Department of Radiology Tangdu Hospital Air Force Medical University Xi'an 710032 China; ^2^ Health Science Center Ningbo University Ningbo 315211 China; ^3^ State Key Laboratory of Electrical Insulation and Power Equipment Centre for Plasma Biomedicine, School of Electrical Engineering Xi'an Jiaotong University Xi'an 710049 China; ^4^ Department of Geriatric Medicine The Affiliated Hospital of Southwest Medical University Luzhou 646000 China; ^5^ Department of Orthopedic Surgery Shanghai Sixth People's Hospital Affiliated to Shanghai Jiao Tong University School of Medicine Shanghai 200233 China; ^6^ Shenzhen Grubbs Institute and Department of Chemistry Southern University of Science and Technology Shenzhen 518055 China; ^7^ The Second Affiliated Hospital of Zhejiang Chinese Medical University Hangzhou 310000 China; ^8^ Department of Anesthesiology the First Medical Center of Chinese PLA General Hospital Beijing 100853 China; ^9^ State Key Laboratory for Manufacturing Systems Engineering International Joint Laboratory for Micro/Nano Manufacturing and Measurement Technologies School of Instrument Science and Technology Xi'an Jiaotong University Xi'an 710049 China; ^10^ CAS Key Laboratory of Nanosystem and Hierarchical Fabrication CAS Center for Excellence in Nanoscience National Center for Nanoscience and Technology Beijing 100190 China; ^11^ School of Science and Engineering, Shenzhen Institute of Aggregate Science and Technology The Chinese University of Hong Kong Shenzhen (CUHK‐Shenzhen) Guangdong 518172 China; ^12^ Department of Plastic Surgery and Regenerative Medicine Fujian Medical University Union Hospital Fuzhou 350001 China; ^13^ Department of Medical Engineering Xinqiao Hospital Army Medical University Chongqing 400037 China; ^14^ Department of Pediatric Orthopaedics Xinhua Hospital Affiliated to Shanghai Jiao Tong University School of Medicine Shanghai 200092 China

**Keywords:** bacterially infected burns, bioelectrical stimulation, charge‐injected hydrogel, electrets, fibrosis inhibition, immune modulation, scar‐free healing

## Abstract

In this study, an electret‐inspired, charge‐injected hydrogel called QOSP hydrogel (QCS/OD/SDI/PANI/PS/Plasma) that promotes scar‐free healing of bacteria‐infected burns through bioelectrical stimulation and immune modulation, is presented. The hydrogel, composed of quaternized chitosan (QCS), oxidized dextran (OD), sulfadiazine (SDI), polystyrene (PS), and polyaniline nanowires (PANI), forms a conductive network capable of storing and releasing electric charges, emulating an electret‐like mechanism. This structure delivers bioelectrical signals continuously, enhancing wound healing by regulating immune responses and minimizing fibrosis. In a mouse model of second‐degree burns infected with *Staphylococcus aureus* (SA) and *Pseudomonas aeruginosa* (PA), the hydrogel accelerates wound healing by 32% and reduces bacterial load by 60%, significantly inhibited scar formation by 40% compared to controls. QOSP hydrogel modulates the Th1/Th2 immune balance toward a Th1‐dominant antifibrotic state through quaternized chitosan, thereby reducing collagen deposition by 35%. Electro‐dielectric characterization reveals a dielectric constant of 6.2, a 34% improvement in conductivity (3.33 × 10^−5^ S/m) and a 30 °C increase in thermal stability. Proteomic analysis highlights a 50% down‐regulation of pro‐inflammatory and pro‐fibrotic pathways, suggesting a controlled immune response conducive to scar‐free healing. This study underscores the potential of bioelectrically active hydrogels as a novel approach for treating infected wounds prone to scarring.

## Graphical abstract

This study presents an electret‐inspired charge‐injected self‐powered hydrogel (QOSP) for the treatment of second‐degree burn wounds infected with *Staphylococcus aureus* and *Pseudomonas aeruginosa*. The hydrogel effectively eliminates bacteria and enhances cell proliferation through electrical stimulation, thereby accelerating wound healing. Additionally, it regulates the immune response, reduces fibrosis, and promotes scar‐free healing through the incorporation of quaternized chitosan, offering a novel approach to wound treatment.

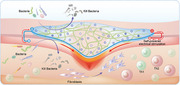

## 1 Introduction

Wound healing, particularly in cases of severe burns, poses a significant challenge in clinical medicine due to the complexity of the healing process and the frequent complications associated with bacterial infections.^[^ ^1^ ^]^ Burns, especially second‐degree and more severe cases, cause extensive tissue damage, resulting in a disrupted skin barrier that delays the natural healing process. Infected burn wounds not only hinder regeneration, but also increase the risk of excessive scarring and fibrosis.^[^ ^2^ ^]^ Dysregulated immune response leads to chronic inflammation, resulting in excessive collagen deposition and scar formation.^[^ ^3^, ^4^ ^]^ Scarring restricts normal wound closure, as well as causes pain and increased sensitivity, making wound healing more challenging.^[^ ^5^ ^]^ Traditional wound dressings, such as gauze and film‐based materials, primarily serve as passive barriers, protecting the wound from external contaminants. While effective in preventing further bacterial invasion, these conventional dressings do little to actively promote tissue regeneration or to deal with the fundamental problems such as infection, inflammation, or immune dysregulation. Moreover, they often fail to prevent the excessive collagen deposition that leads to hypertrophic scarring, which can compromise the skin' s elasticity and post‐healing appearance.^[^ ^6^ ^]^ Thus, there is an urgent need for advanced wound care strategies that not only shield the wound, but also actively promote faster and more effective healing.

Recent advancements in biomaterials, particularly hydrogels, have shown great potential in improving wound healing outcomes.^[^ ^7^, ^8^, ^9^, ^10^, ^11^, ^12^ ^]^ Hydrogels are valued for their ability to create a moist wound environment, which is conducive to tissue repair and regeneration.^[^ ^13^, ^14^ ^]^ Furthermore, hydrogels can be loaded with therapeutic agents, such as antibiotics, growth factors, or bioactive molecules, allowing for sustained and targeted delivery to the wound site.^[^ ^15^, ^16^ ^]^ This localized delivery helps enhance the healing process by addressing infection and supporting tissue growth. Despite these advantages, most conventional hydrogels are inherently passive materials. They do not actively modulate biological processes at the wound site or influence the immune response to prevent fibrosis and scarring. Given the significant roles that inflammation, immune dysregulation, and bacterial infections play in wound healing, the lack of active modulation on these mentioned factors in traditional hydrogels limits their effectiveness, particularly in treating complex wounds such as infected burns. This limitation reduces their effectiveness in treating complex wounds, particularly bacterially infected burns where active intervention is crucial.

One promising approach to actively enhance wound healing is bioelectrical stimulation.^[^ ^17^, ^18^ ^]^ Bioelectricity plays a fundamental role in the body's natural healing processes by influencing key cellular functions, such as cell migration, proliferation, and differentiation.^[^ ^19^ ^]^ Electrical signals generated by the body are critical during the early stages of wound healing, guiding immune cells to the injury site and promoting the formation of new tissue.^[^ ^20^ ^]^ Inspired by the critical role of bioelectricity in these processes, researchers have begun exploring the use of self‐powered biomaterials that mimic or enhance these natural electrical signals.^[^ ^21^, ^22^ ^]^ These bioelectroactive materials have the potential not only to promote cell proliferation and tissue regeneration, but also to reduce infection‐related complications, thereby achieving scar‐free healing.

In recent years, nanomaterials such as carbon nanotubes (CNTs) and graphene have attracted increased attention in biological research. CNTs are attractive materials for tissue engineering because of their diverse electrical, mechanical, and surface properties. CNTs have been shown to aid the epithelialization process in wound healing. CNTs can be functionalized with a variety of pharmacologically active compounds/molecules to increase their performance in terms of drug delivery, activity, and controlled release. E.g., a study reported the generation of 3T3 cell‐based engineered connective tissue (ECT) using different concentrations of functionalized CNTs (f‐CNTs), and the results showed a significant reduction in tissue fibrosis and matrix porosity, indicating that carbon nanotube (CNT)‐collagen composites have attracted widespread attention from researchers.^[^ ^23^ ^]^ One of the most researched carbon‐based nanomaterials is graphene. Graphene oxide has been shown in recent research to support tissue and cell regeneration. Because of its superior conductivity, reduced graphene oxide is more frequently selected than graphene oxide. Graphene oxide has angiogenic properties and can be used in wound dressing patches to deliver and release active agents for wound healing.^[^ ^24^ ^]^ These studies indicate that carbon nanotubes and graphene materials are now being used in preclinical trials for tissue repair, indicating significant potential.

Electrets, materials capable of retaining quasi‐permanent electric charges, have emerged as a promising tool for designing advanced wound dressings. Electrets have long been used in various applications, including sensors and electronic devices, due to their ability to store and release electric charges in a controlled manner.^[^ ^25^ ^]^ Leveraging this property for wound care, electret‐inspired materials can provide continuous bioelectrical stimulation at the wound site, actively influencing cellular processes that accelerate healing. By harnessing the principles of charge storage and controlled release, electret‐inspired materials offer a new avenue for designing wound dressings that surpass traditional passive barriers.^[^ ^26^, ^27^ ^]^ In this context, we have developed an electret‐inspired charge‐injected hydrogel (QOSP hydrogel), specifically designed to treat bacterially infected burns. This hydrogel mimics the electret characteristics by storing and releasing electric charges, providing continuous bioelectrical stimulation that supports healing while reducing the risk of infection and scarring.

The QOSP hydrogel is synthesized using quaternized chitosan (QCS), oxidized dextran (OD), sulfadiazine (SDI), polystyrene (PS), and polyaniline (PANI) nanowires, with each component selected for its specific properties. A unique feature of this system is the post‐synthesis charge injection using a semiconductor plasma process, which enhances its bioelectrical functionality.^[^ ^16^, ^28^ ^]^ In this system, QCS and OD were cross‐linked via Schiff base reaction to form a hydrogel. Chitosan has been proven in many investigations to behave as an immunoadjuvant, increasing Th1‐biased immune responses.^[^ ^29^, ^30^, ^31^, ^32^ ^]^ E.g., Wu et al. published a study that showed that positively charged chitosan‐based nanocomplexes can enhance the immunological effects of Th1 responses by modulating dendritic cell differentiation.^[^ ^29^ ^]^ Our design exploits this property of chitosan to skew the immune response toward a Th1 phenotype, thereby exerting its role in modulating scarring in burn wounds. SDI, a common topical antibiotic for burn wounds,^[^ ^33^ ^]^ was incorporated into the QCS‐OD hydrogel. This not only enhanced its antibacterial activity against local bacteria in burn wounds, but also improved the hydrogel's strength via the Schiff base reaction between SDI and OD. Polystyrene and polyaniline nanowires were incorporated to form a highly conductive network within the hydrogel, enabling efficient charge storage and release.^[^ ^34^ ^]^ This conductive network allows the hydrogel to deliver bioelectrical stimulation to the wound site continuously, enhancing cellular activities such as migration, proliferation, and differentiation. This design enables the charge‐injected QOSP hydrogel to simultaneously exert the effects of bioelectric stimulation and immunomodulation, thereby achieving scar‐free healing of bacterially infected burn wounds.

This study seeks to explore the effectiveness of QOSP hydrogel in promoting scar‐free healing, particularly in burns complicated by infections with *Staphylococcus aureus* (SA) and *Pseudomonas aeruginosa* (PA), a common and challenging bacterial pathogen in burn wounds. We propose that QOSP hydrogels improve antimicrobial activity via a charge injection mechanism and help modulating immune responses. The inherent properties of quaternized chitosan promote a microenvironment favorable for scar‐less tissue healing. By encouraging a shift from a pro‐fibrotic to an anti‐fibrotic immune response, we aim to significantly reduce scar formation while speeding up the overall healing process. To validate these hypotheses, we conducted a comprehensive series of comprehensive in vitro and in vivo experiments, evaluating the hydrogel' s capacity to enhance healing, lower bacterial load, and limit scarring. We believe this approach has the potential to lead to a breakthrough in wound care by providing a highly effective treatment option for complex wounds prone to infection and excessive fibrosis, such as diabetic foot ulcers, pressure sores, chronic ulcers, post‐surgical infections, and traumatic skin injuries. Ultimately, this work could pave the way for the development of a new generation of smart, bioactive wound dressings, offering a significant advancement over the current standard of care for burn wounds and other challenging clinical cases.

## 2 Results and Discussion

### 2.1 Fabrication and Electro‐Physicochemical Characterization of QCS/OD/SDI/PANI/PS/Plasma (QOSP) hydrogels

**Figure 1:**
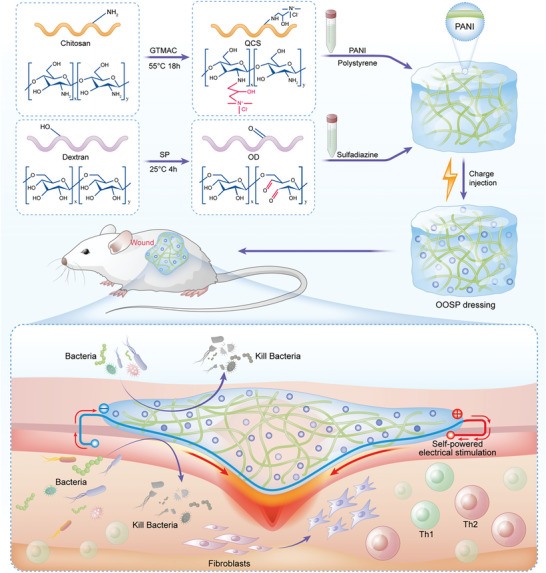
The preparation process of QOSP hydrogel includes the preparation of quaternized chitosan (QCS) and OD, the incorporation of polystyrene (PS) and polyaniline nanowires (PANI), and then the injection of charge into the QOSP hydrogel to obtain a new self‐powered hydrogel dressing, which is then applied to the skin wounds of bacterially infected second‐degree burn mice to provide electrical stimulation. QOSP hydrogel promotes scar‐free wound healing mainly by inducing bioelectricity, sterilization, and immune regulation to inhibit excessive fibrosis.

The charged QOSP hydrogel dressing adopts a composite structure design, as shown in **Figure** **1**. The basic hydrogel is synthesized by the Schiff base reaction between QCS and OD, and SDI is added to further promote cross‐linking and enhance the overall mechanical and structural integrity of the material. At the same time, conductive polyaniline (PANI) nanowires and polystyrene are added to the hydrogel matrix, and charge injection is carried out using semiconductor process plasma treatment. The uniform distribution of polystyrene helps to achieve uniform and long‐term storage of charges. This charge, together with the conductive PANI nanowires, establishes a conductive pathway network that can achieve continuous bioelectric stimulation at the wound site, thereby promoting cell proliferation and differentiation. In addition, QOSP hydrogel has good antibacterial function and immunomodulatory ability and can support the formation of an anti‐fibrotic environment for burn wounds, which further enhances the potential for promoting healing and helps to achieve scar‐free repair of the wound.

**Figure 2:**
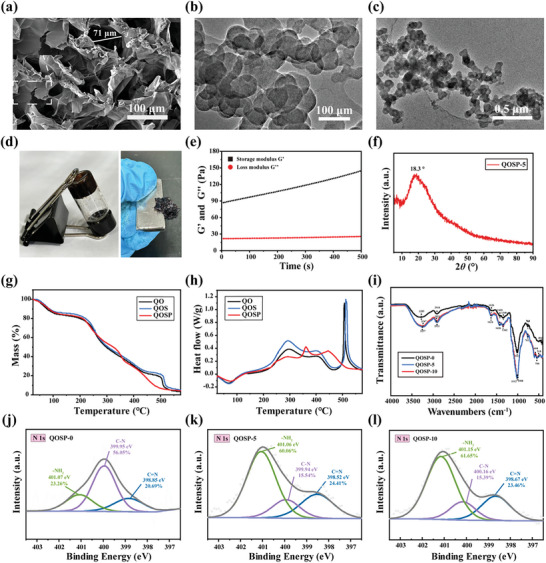
Characterizations of QOSP hydrogels. a) SEM images of the QOSP hydrogel after lyophilization, magnified 1000×. b,c) TEM images of the QOSP hydrogel after lyophilization. d) Schematic diagram of QOSP hydrogel. Movies 1–3, Supporting Information show the adsorption capacity of the charged hydrogel e) Rheological properties of QOSP‐5 hydrogels. f) XRD analysis of QOSP‐5 hydrogels. g) TGA profiles of QO, QOS, and QOSP hydrogels. h) DSC of QO, QOS, and QOSP hydrogels. i) FTIR spectra of QOSP‐0, QOSP‐5, and QOSP‐10 hydrogels. j,k) XPS results of QOSP‐0, QOSP‐5, and QOSP‐10 hydrogels.

The morphology of the freeze‐dried QOSP‐5 hydrogels was characterized by scanning electron microscopy (SEM), as shown in **Figure** **2**a. High‐magnification images revealed a highly ordered porous structure, with polyaniline nanowires well‐distributed throughout the hydrogel matrix. The pore size is ≈71 µm, providing a large surface area for charge conduction channels and storage layers. Individual particles with diameters as small as 45 nm are visible. Charge injection facilitates uniform dispersion of the polyaniline nanowires, which form an interconnected network crucial for conductivity and bioelectrical stimulation therapy in wound healing (Figure 2b,c). The three hydrogels are represented by: QCS/OD (QO), QO/SDI/PANI/PS (QOS), QOS/Plasma (QOSP). More SEM images of five hydrogels are provided in the Figures S1,S2 (Supporting Information).

Figure 2d shows the physical appearance of the QOSP hydrogel, highlighting its opaque, gel‐like texture and good adhesion. Figure 2e shows the rheological behavior of the QOSP‐5 hydrogel over 500s, where the storage modulus G′ and loss modulus G″ are plotted. The storage modulus G′ (represented by the black line) continues increasing throughout the test period, indicating the solid‐like behavior of the hydrogel and its ability to store energy upon deformation. The storage modulus G’ starts at ≈60 Pa and then develops to slightly greater than 140 Pa. It is worth noting that the storage modulus G’ of QOSP‐5 is significantly higher than that of QO and QOS as shown in Figure S3 (Supporting Information), which may be related to the enhanced mechanical strength due to the addition of PANI and PS. Charge injection further increases this property, as reflected in the higher G’ (from 135 Pa to 150 Pa) of QOSP‐10. In sharp contrast, the loss modulus G″ (indicated by the red line) of the five hydrogels remains almost unchanged and is significantly lower than G′, hovering ≈30 Pa. The difference between G′ and G″′ accentuates the advantage of elastic properties over viscous properties, highlighting the promise of QOSP‐5 hydrogels in applications with high elasticity and low energy dissipation.

Additionally, the X‐ray Diffraction (XRD) pattern of the QOSP‐5 hydrogel after charge injection (Figure 2f) shows prominent diffraction peaks at 18.3°, indicating a more precise crystal orientation than the two peaks of QO at 17.7° and 28.8° (Figure S4, Supporting Information). As the charge injection duration increases, the peak positions of QOSP‐5 and QOSP‐10 become more concentrated and sharper, showing that continuous charge injection promotes crystallinity and organizes the structure. The charge injection process likely aligns the PANI chains and enhances doping effects, promoting specific crystalline planes. These changes improve both the structural stability and electrical properties of the hydrogel, making it suitable for electrochemical applications.^[^ ^35^, ^36^ ^]^

Figure 2g,h presents the thermogravimetric analysis (TGA) and differential scanning calorimetry (DSC) curves for QO, QOS, and QOSP hydrogels. The TGA results show thermal degradation temperatures of ≈280 °C for QO, 290 °C for QOS, and 310 °C for QOSP, indicating that the incorporation of sulfadiazine, polystyrene, and polyaniline enhances thermal stability. The hydrogel's thermal stability was greatly enhanced due to multiple cross‐links between components, slowing down the thermal degradation. The DSC scans reveal that QO has a significant endothermic peak at ≈300 °C, representing the breakdown of quaternized chitosan and OD. In contrast, QOS shows a slightly higher intensity peak at the same temperature, reflecting the stabilization effect of sulfadiazine. The QOSP hydrogel exhibits a broader endothermic peak and an additional exothermic peak at ≈500 °C, corresponding to the decomposition of polystyrene and polyaniline. These components not only enhance the mechanical strength of the hydrogels, but also improve their thermal behavior, resulting in better stability and more distinct phase transitions.^[^ ^37^, ^38^, ^39^ ^]^

To gain a comprehensive understanding of the molecular structure changes within the QOSP hydrogel after charge injection, FTIR testing was conducted, as illustrated in Figures 2i and S5 (Supporting Information). The samples QOSP‐0, QOSP‐5, and QOSP‐10 represent no high‐pressure charge injection treatment, high‐pressure treatment for 5 min, and high‐pressure treatment for 10 min, respectively. The imine (C═N) bond's distinctive peak (1640–1690 cm^−1^) indicates effective Schiff base formation. As we advance from QOSP‐0 to QOSP‐10, the transmittance of this peak falls somehow, indicating that high‐voltage electric charge injection does not impede but stimulate Schiff base cross‐linking.^[^ ^40^ ^]^ Notably, distinct absorption peaks at 3285 cm^−1^ and 3257 cm^−1^, corresponding to O‐H and N‐H stretching vibrations respectively. As the treatment duration increases, the transmittance of these peaks diminishes, revealing that charge injection can significantly enhance the presence of polar functional groups (such as hydroxyl and amino groups), thereby increasing the hydrogel's hydrophilicity and reactivity. Additionally, the peaks at 2918 cm^−1^ and 2921 cm^−1^, attributed to C─H stretching vibrations, also change, indicating a rearrangement of molecular chains in the polystyrene and polyaniline components. Significant alterations were detected at the peaks at 1626 cm^−1^ and 1624 cm^−1^, corresponding to C═O and C═C stretching vibrations. These stretching vibrations imply a rearrangement of molecular chains and modifications in the conjugated system or carbonyl groups, likely due to several mechanisms: electric field‐induced dipole realignment, charge migration, and ionic conduction, local thermal effects from current‐induced heating, electrochemical reactions, and charge‐induced polymerization. These processes contribute to an overall enhancement of the hydrogel's dielectric functionality, making it suitable for bioelectronic interfaces, where its structural and chemical adaptability under electrical stimulations is crucial.^[^ ^41^, ^42^, ^43^ ^]^

Figure 2j–l depicts the XPS results of QOSP‐0, QOSP‐5, and QOSP‐10, respectively. The data shows three distinct peaks for nitrogen species: ‐NH₂ at 401.07 eV, C─N at 399.95 eV, and C═N at 398.85 eV. In QOSP‐0, the relative intensities of these peaks are 23.26%, 56.05%, and 20.69%, respectively. After 5 min of electric field treatment (QOSP‐5), the relative intensity of ‐NH₂ increased by 60.06%, C─N by 15.54%, and C═N by 24.41%. After 10 min of treatment (QOSP‐10), the intensities adjusted to ‐NH_2_ 61.65%, C─N 15.39%, and C═N 23.46%. The electric field treatment increased the number of ‐NH₂ groups while reducing the number of C─N and C═N groups. These findings suggest that the electric field treatment successfully affects the chemical structure of the hydrogel and enhances the polarity of the material through the introduction of surface functional groups, which may introduce free charges or change its charge distribution, further corroborating the morphological findings (Figure S2, Supporting Information also supports this view).

### 2.2 QOSP Electro‐Dielectric Characterization

**Figure 3:**
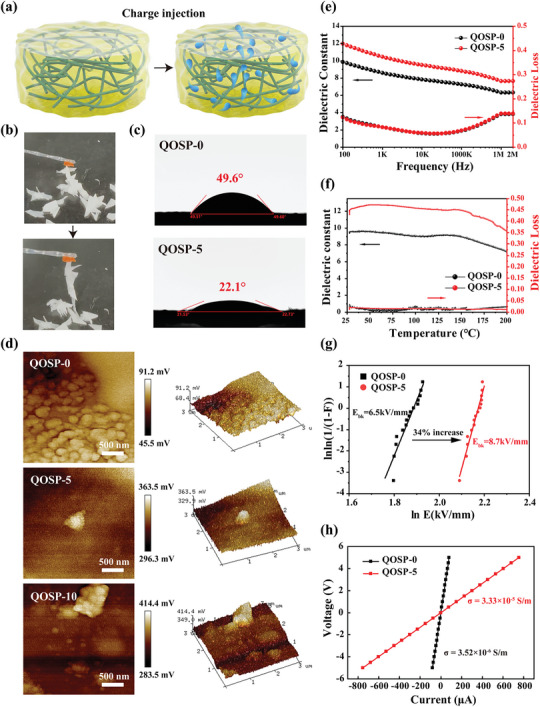
a) Schematic diagram of the coupled plasma etching charge injection process. b) Under the electrostatic force, QOSP hydrogels can firmly absorb the shredded paper. c) Water contact angles of QOSP‐0 (*θ*=49.6°) and QOSP‐5 (*θ*=22.1°) hydrogels, respectively. d) Scanning Kelvin Probe Microscopy (SKPM) surface potential maps of QOSP hydrogels. e) Frequency dependence of the dielectric constant of QOSP‐5 hydrogel at room temperature. f) Temperature dependence of dielectric constant of QOSP‐5 hydrogel at 1 kHz frequency. g) DC dielectric breakdown strength of QOSP‐0 and QOSP‐5 hydrogels with statistical data from 15 samples. h) Electrical conductivity of QOSP‐0 and QOSP‐5 hydrogels (Movie 6, Supporting Information).

**Figure** **3**a shows the charge injection process by the Capacitive Coupling Plasma (CCP) system and more detailed information can be found in Supporting Information 2. Figure 3b,c, the improvement of electrostatic adsorption ability and the improvement of hydrophilicity in contact angle experiment prove the previous morphological findings. To further understand the chemical changes on the surface of the QOSP hydrogels after charge injection, X‐ray Photoelectron Spectroscopy (XPS) was performed. The C 1s spectra (Figure S6a–c, Supporting Information) revealed significant increases in the C─O bond content after 5 min of plasma treatment (QOSP‐5), with the C─O peak rising to 39.4%, indicating accelerated oxidation. Similarly, the O 1s spectra (Figure S6d–f, Supporting Information) demonstrated a substantial increase in hydroxyl groups (‐OH) from 35.98% in QOSP‐0 to 63.77% in QOSP‐5 and 70.66% in QOSP‐10. These changes suggest enhanced surface oxidation and hydrophilicity, which are consistent with the improved bioelectrical and conductive properties of the hydrogels. The increase in surface oxygen content and the formation of hydroxyl groups contribute to a more hydrophilic and reactive surface, enhancing both the electrical conductivity and biological interactions of the hydrogel. To explore the self‐powered properties of QOSP hydrogel, surface potentials of three different hydrogel samples were measured using a scanning Kelvin probe microscope (SKPM), as shown in Figure 3d. The surface potential of QOSP‐0 ranges from +45.5 mV to +91.2 mV, indicating minimal change without high‐pressure treatment. This stability is likely due to the inherent positive charge of quaternary ammonium chitosan. Notably, the surface potentials of QOSP‐5 and QOSP‐10 significantly increased, ranging from +296.3 mV to +363.5 mV and +283.5 mV to +414.4 mV, respectively, indicating substantial charge accumulation and potential hotspots.^[^ ^44^ ^]^ Furthermore, the material's components, such as QCS, which contains cationic groups, can help stabilize the injected positive charges. The conductive PANI nanowires within the hydrogel also facilitate the transfer and retention of these positive charges, enhancing the material's ability to store and maintain the injected charge. To assess the surface charge retention of QOSP hydrogels, the surface potential attenuation curves of QOSP‐0, QOSP‐5, and QOSP‐10 were measured (Figure S8, Supporting Information). QOSP‐5 and QOSP‐10 hydrogels exhibited significantly improved charge retention compared to QOSP‐0, as demonstrated by their slower surface potential decay over time.^[^ ^45^ ^]^ This enhanced charge retention is attributed to the improved surface oxidation and charge distribution following plasma treatment. The ability to maintain surface potential over time is crucial for providing continuous bioelectrical stimulation at the wound site, enhancing cellular proliferation and tissue regeneration (Figure S12, Supporting Information). The reasons we use QSCP‐5 in experiments are detailed in Supporting Information 1.4.^[^ ^46^ ^]^

Figure 3e presents the dielectric constant (ε’) and dielectric loss (tan δ) as a function of frequency for two samples: QOSP‐0 and QOSP‐5. The dielectric constant for both samples decreases as the frequency increases, which is typical behavior for polymeric dielectrics due to the decreasing ability of dipoles to align with the rapidly changing electric field at higher frequencies. At 1 kHz, the QOSP‐0 dielectric constant starts at ≈6.5 and gradually decreases to ≈3.2 by 1 MHz. The dielectric loss for this sample also shows a decreasing trend, starting from ≈0.46 at 1 kHz and reducing to near 0.06 at 1 MHz. Post charge injection, the QOSP‐5 dielectric constant begins at ≈6.2 at 1 kHz and follows a similar decreasing trend, reaching ≈3.0 at 1 MHz. However, the dielectric loss is consistently lower than that of QOSP‐0 across the frequency range, starting from 0.44 at 1 kHz and diminishing to ≈0.05 at 1 MHz. The lower dielectric loss in QOSP‐5 suggests improved electrical insulation properties post high‐voltage treatment, possibly due to better polymer chain alignment and reduced ionic mobility within the hydrogel structure. Figure 3f depicts how the dielectric properties of QOSP‐0 and QOSP‐5 change with temperature, ranging from 25 °C to 175 °C. Both dielectric constant and loss are observed to increase with temperature.

For QOSP‐0, the dielectric constant rises from ≈3.4 at 25 °C to ≈4.2 at 175 °C, showing a moderate increase. The dielectric loss shows a more significant increase, starting from 0.22 at 25 °C and reaching up to 0.35 at 175 °C, which suggests increased ionic mobility at higher temperatures. For QOSP‐5, the dielectric constant begins at ≈3.3 at 25 °C and increases to ≈3.8 at 175 °C. The dielectric loss, starting from 0.20 at 25 °C, climbs slightly to 0.25 at 175 °C. The generally lower values of dielectric loss compared to QOSP‐0 indicate that the high‐voltage treatment may have enhanced the thermal stability of the hydrogel, reducing the increase rate of ionic mobility with temperature. The observations from Figure 3e,f indicate that high‐voltage treatment not only modifies the electrical properties of QOSP hydrogels by enhancing the uniformity and alignment of polymer chains, but also appears to affect their thermal behavior, reducing the thermal dependency of dielectric loss. This could make QOSP‐5 more suitable for applications when electrical performance at varying temperatures is critical.

Dielectric breakdown strength is crucial for assessing a material's dielectric properties.^[^ ^47^ ^]^ A higher breakdown strength indicates better resistance to electrical failure, enhancing charge storage and stability under high electric fields. Figure 3g illustrates the direct current (DC) breakdown data for QOSP‐0 and QOSP‐5, fitting a Weibull distribution. The average DC breakdown strength for QOSP‐0 is 6.5 kV mm⁻^1^, while for QOSP‐5, it is significantly higher at 8.7 kV mm⁻^1^, with a 34% improvement. This increase is attributed to molecular rearrangement induced by the high‐voltage electric field, which redistributes charges within the hydrogel matrix, leading to a more uniform charge distribution and reduced local electric field strength. Generally, semiconductor hydrogels have lower DC breakdown strength than other semiconductor materials due to high water content, ionic conductivity, and interfacial effects. Additionally, the conductivity of QOSP‐5 hydrogel is higher than that of QOSP‐0, increasing from 3.52 × 10^−6^ S m⁻^1^ to 3.33 × 10^−5^ S m⁻^1^, as shown in Figure 3h, due to improved network connectivity and interfacial effects. These results are consistent with previous SKPM analyses, fully confirming the enhanced charge injection and molecular rearrangement due to high‐voltage electric field treatment, resulting in increased conductivity and dielectric breakdown strength.

The self‐healing ability of the QOSP hydrogels was also evaluated, as it is a critical factor for their durability and long‐term application in wound care. The self‐healing properties of QOSP‐0, QOSP‐5, and QOSP‐10 hydrogels are shown in Figure S7a–c (Supporting Information). QOSP‐5 hydrogels exhibited superior self‐healing behavior compared to QOSP‐0, attributed to the improved cross‐linking density and enhanced charge distribution following charge injection. As shown in Figure S9 (Supporting Information), the viscosity of QOSP‐5 hydrogels decreased with the temperature increase, indicating a temperature‐dependent fluidity. This behavior suggests that the hydrogel maintains its mechanical integrity across a range of physiological temperatures, which is important for its application in wound healing when temperature fluctuations may occur. The thermally stimulated depolarization current (TSDC) analysis was performed to further evaluate the thermal stability and charge storage behavior of QOSP‐0 and QOSP‐5 hydrogels. As shown in Figure S11 (Supporting Information), QOSP‐0 exhibited a sharp depolarization peak ≈90 °C, indicating a significant release of stored charges at this temperature, which suggests structural changes within the hydrogel matrix. In contrast, QOSP‐5 showed a wider but smaller depolarization peak ≈140 °C, indicating an increased cross‐linking density and more uniform charge distribution following charge injection. This shift in the TSDC curve for QOSP‐5 correlates with improved thermal stability and enhanced electrical functionality, making it more suitable for applications requiring stable performance under varying thermal conditions.

### 2.3 Biocompatibility and Antimicrobial Properties of QOSP

**Figure 4:**
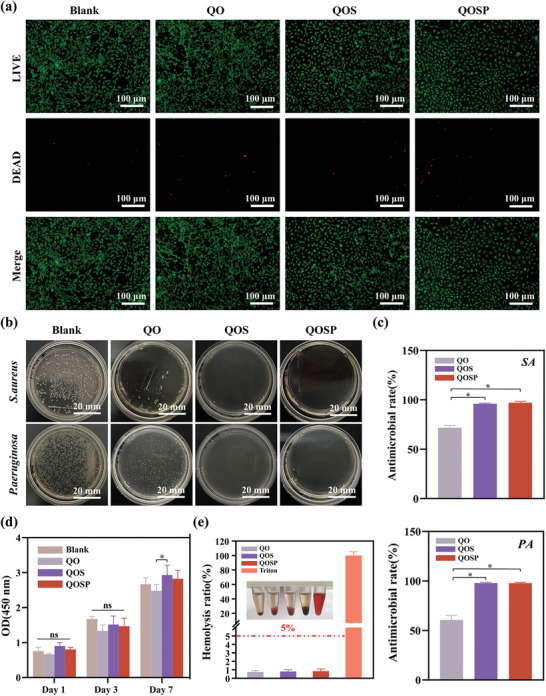
Studies of the biocompatibility and anti‐bacterial activity of the QOSP hydrogels. a) Live/Dead staining of fibroblasts co‐cultured with the hydrogels for 7 days; green represents live cells, while red represents dead cells. Cells cultured on a tissue culture plate (TCP) were used as blank. b) Representative photographs of the antimicrobial effect of QOSP hydrogels against SA and PA bacteria for 24 h. c) Quantitative analysis of the SA and PA colonies incubated with each hydrogel for 24 h (* *p* < 0.05). d) Cell proliferation analysis of fibroblasts co‐cultured with the QOSP hydrogels for 1, 3, and 7 days. The data were presented as mean ± SD (n=3,* *p* < 0.05). e) Hemolysis ratio of the QOSP hydrogels.

Excellent biocompatibility is the fundamental requirement for medical dressings, serving as the essential characteristic that facilitates their use as an interface between tissue and sensors. Cytocompatibility and haemocompatibility are two of the most common ways to assess hydrogel materials. We assessed the biocompatibility of the hydrogels using fibroblasts (NIH/3T3) as the cell model by CCK‐8 and Live/Dead staining. The results showed that QO, QOS, and QOSP hydrogels exhibited excellent cytocompatibility after 1, 3, and 7 days of co‐culture, comparable to that of the tissue culture plates (Blank group) (**Figure** **4**d). Live/Dead staining analysis revealed that the hydrogels were highly biocompatible, with a low percentage of dead cells after 7 days of co‐culture (Figure 4a). Furthermore, the hemolysis test showed that contact with these hydrogels caused minimal rupture of red blood cells (well below the safety threshold of 5% hemolysis) (Figure 4e).

Before complete epithelialization, burn injuries are particularly prone to infections from invasive microbes, predominantly *Staphylococcus aureus* (SA) and *Pseudomonas aeruginosa* (PA).^[^ ^16^ ^]^ The release behavior of SDI from the QOSP‐5 hydrogels was evaluated under physiological and slightly acidic conditions to simulate infected wound environments. As shown in Figure S13a (Supporting Information), SDI release was faster at pH 5.5 compared to pH 7.4, indicating that the hydrogel is capable of providing a more rapid drug release in acidic, infection‐prone environments, enhancing its antibacterial efficacy. Plate counting experiments revealed the bactericidal capabilities of hydrogel against co‐cultured bacteria, suggesting promising applications in managing infected burn wounds (Figure 4b,c). Additionally, the degradation profiles of QOSP‐5 hydrogels (Figure S13b, Supporting Information) demonstrated that the hydrogels degraded more quickly in acidic conditions, which is beneficial for applications where faster degradation and drug release are required for treating infected wounds. The antimicrobial properties of the QOSP‐5 hydrogel were further confirmed by assessing the bacterial growth curves for SA and PA after treatment with the hydrogel. As shown in Figure S14 (Supporting Information), the growth of both SA and PA was significantly inhibited at various concentrations of the hydrogel, demonstrating the strong antibacterial activity of QOSP‐5. The results suggest that the combination of SDI release and the intrinsic properties of the hydrogel matrix work synergistically to suppress bacterial growth, which is critical for preventing infection in wound healing applications.

### 2.4 In Vivo Effect of QOSP

**Figure 5:**
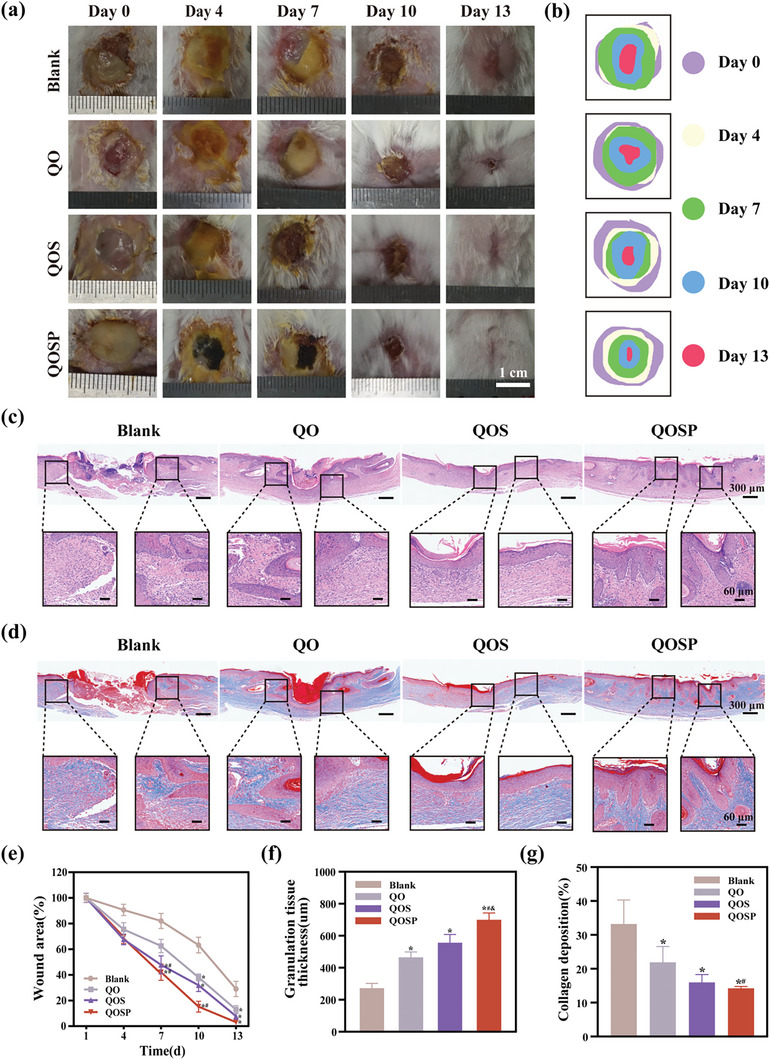
a) Representative images of the wounds in the groups of QO, QOS, QOSP hydrogels, and the blank group without hydrogel dressing treatment from day 0 to day 13. b) Wound traces at different time points. HE (c) and Masson (d) staining on day 13 of the newly regenerated skin tissues for each group. e) Quantitative data of wound closure. f) Quantitative data of granulation tissue thickness on day 13, which is obtained by measuring the epithelialization distance in HE‐stained tissue slices. g) Quantitative data on collagen deposition obtained from Masson‐stained tissue slices measured by Imagej.

Burns are a prevalent health issue, and second‐degree and more severe burns are frequently associated with serious bacterial infections and proliferative scarring. Hence, a model of a burn wound infected with bacteria was utilized to evaluate the efficacy of the QOSP‐5 dressings in promoting wound healing. Once the mice were completely anesthetized, their back skin was contacted with a circular aluminum rod heated to 180 °C for 10 s to form a burn wound model. After the wound cooled, we spread 50 µL of a mixed bacterial solution (50% SA and 50% PA, both at a concentration of 1 × 10^7^ CFU mL⁻^1^) onto the wound surface and covered it with a Tegaderm™ film to create an infected burn model. Two days later, we confirmed the successful establishment of the infected burn model. The wounds in each group received different hydrogel treatments and were covered and secured with Tegaderm™ film. The Blank group was only covered with Tegaderm™ film. After four days of therapy, the wound contraction rate in the Blank group was only 9% due to the severe wound infection, but the wound contraction rates in the QO, QOS, and QOSP‐5 groups were 31%, 32%, and 32%, respectively (**Figure** **5**a,b,e). As the treatment period progressed, the wounds in each group showed increased contraction. Following 7 days of treatment, the wounds in the Blank and QO groups were still significantly infected, exhibiting wound contraction rates of 16% and 32%, respectively, compared to 52% and 57% in the QOS and QOSP‐5 groups.

After 10 days of therapy, all groups' wounds constricted significantly, however the Blank group's contraction rate remained only 63%, while the QO and QOS groups with good antimicrobial qualities contracted at 70% and 68%, respectively. Notably, the QOSP‐5 group experienced a contraction rate of up to 85% on day 10. After 13 days of therapy, all groups' wounds were totally constricted, except for the Blank group, where the wounds were only 88% contracted and scar formation occurred. The continuous bioelectrical stimulation provided by the charge‐injected QOSP hydrogel likely contributed to accelerated wound closure and minimized fibrosis, demonstrating its superiority over traditional hydrogel dressings.

The hematoxylin‐eosin (HE) staining technique is an important approach for histologic evaluation and immunohistochemical inspection, which is required to monitor the progression of wound healing and skin tissue regeneration. To acquire a better understanding of how QOSP series hydrogel accelerates burn wound healing and promotes scarless wound repair, we examined wound tissues on day 13 using HE staining. The findings indicated that the granulation tissue thickness in the Blank group was limited to 272 µm. In contrast, the granulation tissue thickness in the QO, QOS, and QOSP‐5 groups progressively increased, with the QOSP‐5 group achieving the greatest thickness at 699 µm (Figure 5c,f). However, while we expect more collagen to speed up wound filling during the proliferation stage, excessive collagen deposition in the latter stages of wound healing may result in hypertrophic scarring. As a result, a reasonable amount of collagen is preferable. Masson staining revealed that the collagen distribution of QOSP‐5 group was similar to that of normal tissue on day 13, resulting in a relatively effective healing; but the edge and center of the wound in the Blank group had prominent blue patches, which was substantially greater than the other groups (*p* < 0.05). This phenomenon indicates a substantial amount of collagen deposition in the Blank group and it may lead to scarring eventually (Figure 5d,g). Compared with the QOS group, the QOSP dressing accelerates cell adhesion due to the surface charge it carries, and promotes cell proliferation, migration, and differentiation by simulating the bioelectricity generated during the natural wound healing process. Unlike general conductive hydrogels, it does not require the application of additional electrical stimulation and can independently conduct electricity and transmit electrical stimulation, which is beneficial to the healing of burn wounds.

### 2.5 Potential Mechanism of QOSP Affecting Burn Wound Healing

**Figure 6:**
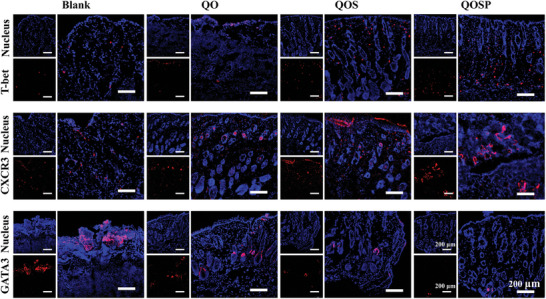
Images of immunofluorescence labeling of the regenerated tissue of wounds stained with T‐bet, CXCR3 and GATA3 on day 13. Scale bar: 200 µm.

**Figure 7:**
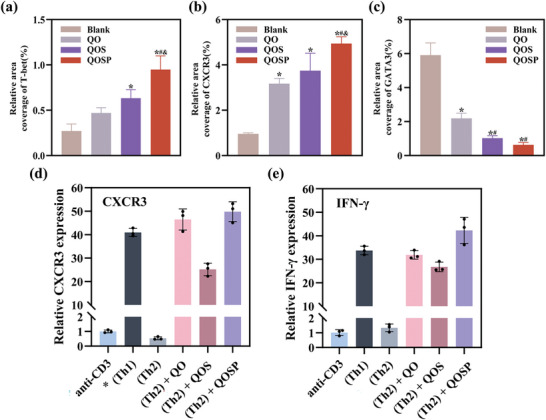
Quantitative analysis of relative area percentage of a) T‐bet, b) CXCR3, and c) GATA3, respectively. Data were shown as mean ± SD (n=3,* *p* < 0.05 when compared with Blank group; ^#^ *p* < 0.05 when compared with QO group; & *p* < 0.05 when compared with QOS group). The relative mRNA expression levels of (d) CXCR3 and e) IFN‐γ in Jurkat E6‐1 cells, analyzed by real‐time qPCR. CXCR3 and IFN‐γ are specific markers of Th1 cells, indicating the expression level of Th1 cells.

Skin scarring remains a hallmark feature after cutaneous burns, where the mechanism of proliferative scarring is often associated with hyperfibrosis. Research shows that various helper T‐cell subtypes significantly influence fibrous deposition in wound healing.^[^ ^2^ ^]^ Th1 cells secrete cytokines like IL‐2, IFN‐γ, and IL‐12, which stimulate fibroblasts to produce collagenase, thereby preventing excessive collagen buildup. On the other hand, Th2 cells induce fibroblast differentiation and reduce collagenase secretion by expressing IL‐4, IL‐5, and IL‐10. During the healing of burn wounds, Th2 cells are more active than Th1 cells, resulting in excessive fibrous deposition, which is a key factor in scar formation. Through immunohistochemistry, we evaluated the effects of different hydrogel dressings on the Th1/Th2 balance in burn wounds during the later stages of healing (day 13). The results (**Figures** **6** and **7**a–c) revealed that in wounds treated with QOS and QOSP hydrogels, the number of Th1 cells (identified by specific antigen T‐bet and CXCR3) was considerably greater than that of Th2 cells (identified by specific antigen GATA3), indicating a shift toward Th1 dominance. In the Blank group, however, GATA3‐positive cells were more prevalent than in other groups, suggesting a Th2 cell predominance, which may explain the extensive scar tissue formation in this group. We proceeded with cell experiments to further study the effects of QOSP hydrogel on the Th1/Th2 balance. We first induced Jurkat cells to polarize toward Th2 to simulate a severely scarred microenvironment. After co‐culturing with different hydrogels, we analyzed the mRNA levels of polarization markers in Jurkat cells. As indicated in Figure 7d,e, all three hydrogel groups significantly enhanced the expression of Th1 polarization markers, while there was no notable impact on Th2 markers (Figure S17a,b, Supporting Information). This indicates that QOSP hydrogel can effectively promote Th1 polarization in lymphocytes, adjust the Th1/Th2 balance, and minimize excessive fibrous deposition and scar formation.

**Figure 8:**
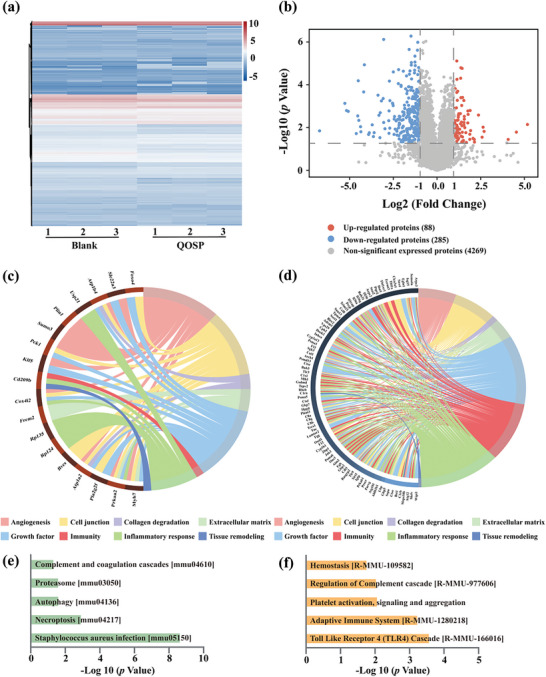
a) Heatmap showing the differences in protein expression levels between the blank and QOSP groups. b) Volcano plot presenting DEPs (log2 FC=1, *p* < 0.05). c) The outcomes of the gene ontology (GO) analysis for up‐regulated proteins. d) The outcomes of the gene ontology (GO) analysis for down‐regulated proteins.) KEGG analysis results of the down‐regulated proteins. f) Reactome analysis results of the down‐regulated proteins.

To further explore the therapeutic mechanism of QOSP hydrogels, label‐free proteomics (LFP) was used to identify the differential expression of proteins (DEPs) between the Blank and QOSP hydrogel groups (Figure S18a, Supporting Information). Overall, 4 642 proteins were discovered in two groups of six samples, with 4 513 being co‐expressed (**Figure** **8**a). The results of principal component analysis (PCA) showed that the component profiles of the two groups differed noticeably, which can be found in Figure S18b (Supporting Information). Compared with the Blank group, 88 proteins were identified as up‐regulated and 285 proteins up‐regulated as down‐regulated in wounds treated with QOSP hydrogel (the criteria for defining differential expression were: adjust *p*‐value < 0.05 and |Log2FC| >1) (Figure 8b). The GO enrichment analysis’ findings revealed that proteins related to cell junction and tissue remodeling were significantly up‐regulated (Figure 8c). In contrast, down‐regulated proteins were predominantly associated with processes related to immunity, inflammatory response, and growth factor (Figure 8d). Detailed identification of the downregulated proteins in the KEGG and Reactome pathways is provided in Table S2 (Supporting Information). Then further investigation was performed by KEGG and Reactome pathway analysis, and we explored the down‐regulated signaling pathways in depth (Figure 8e,f). In the KEGG analysis, we observed a decrease in the “Complement and coagulation cascades” pathway after QOSP hydrogel treatment, indicating reduced inflammation and coagulation. This explains that the wound can enter the healing phase more quickly, since QOSP hydrogel speeds up the resolution of the coagulation and inflammatory phases. The down‐regulation of the “Proteasome” and “Autophagy” pathways suggests a less stressed cellular environment, while the suppression of “Necroptosis” points to decreased inflammation and cell death. Additionally, the down‐regulation of the “Staphylococcus aureus infection” pathway implies potential antimicrobial effects or enhanced immune response, which is consistent with the results we obtained in the antibacterial experiment, indicating that the strong antibacterial effect of the hydrogel effectively improves the wound healing ability. Reactome analysis further highlighted the down‐regulation of hemostasis‐related pathways, suggesting that QOSP hydrogel supports the transition from the hemostatic to the proliferative phase of wound healing. The suppression of the “Complement cascade” and “TLR4” pathways indicates a controlled immune response, essential for minimizing excessive inflammation and promoting tissue repair. Specifically, a number of DEPs significantly affect these pathways. E.g., down‐regulation of Psma1, Psma5, Psmd6, and Psma13 proteins appears to lead to inhibition of the proteasome pathway, whereas down‐regulation of Fgg and Fgb proteins attenuates signaling pathways such as complement and coagulation cascades, hemostasis, platelet activation, signaling and aggregation. Besides, a PPI network analysis was performed to understand the functional significance of up‐ and down‐regulated proteins, which can be found in Figure S18c,d (Supporting Information).

The results of proteomics provide rich biological information and we further explored five core targets using western blot technology, which are shown in Figure S19 (Supporting Information). TLR3 is a pattern recognition receptor that specifically identifies viral RNA, playing a crucial role distinct from the TLR4 pathway. Previous studies have found that skin epithelium requires TLR3 to produce normal inflammation after injury.^[^ ^48^ ^]^ However, excessive inflammatory response is not conducive to the repair of burn wounds. QOSP hydrogel may help control excessive immune response after burns and promote a more balanced and blanklable inflammatory environment by inhibiting TLR3. Reduced Tagln2 expression may affect cytoskeletal reorganization and reduce excessive fibroblast activity, thereby reducing scar formation and fibrosis. Jak3 and Stat2 are key factors in the JAK‐STAT pathway, involved in regulating immune responses and cell growth.^[^ ^49^ ^]^ Their marked down‐regulation suggests that this signaling pathway is blocked, which aids in limiting the production of inflammatory cytokines and hastens the progression of infected burn wounds from the inflammatory to the proliferative phases. MLKL, a pivotal regulator of programed necroptosis, exhibits protective roles against exacerbated inflammatory responses when downregulated. Typically, necroptosis serves as a pro‐inflammatory cell death pathway, mediated by MLKL following its phosphorylation and oligomerization, which compromises membrane integrity leading to cell lysis. Thus, the suppression of MLKL can potentially attenuate cell death in pathophysiological states, thereby preserving tissue integrity and reducing inflammatory cytokine release, which is crucial for the recovery of damaged skin tissues.^[^ ^50^ ^]^

**Figure 9:**
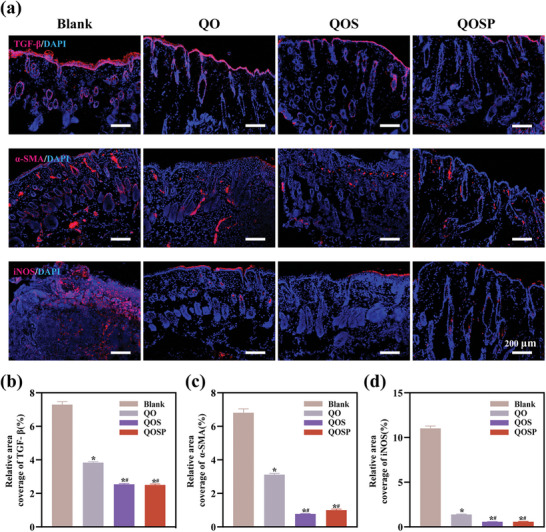
a) Immunofluorescence observation of TGF‐β, α‐SMA and iNOS staining. Scale bar: 200 µm. Data were shown as mean ± SD (n=3,* *p* < 0.05 when compared with Blank group; ^#^ *p* < 0.05 when compared with QO group). Quantitative analysis of relative area percentage of b) TGF‐β, c) α‐SMA and d) iNOS, respectively. Data were shown as mean ± SD (n=3,* *p* < 0.05 when compared with Blank group; ^#^ *p* < 0.05 when compared with QO group; & *p* < 0.05 when compared with QOS group).

Using immunofluorescence technology, we studied the inflammatory indicators and fibrosis conditions in each group of tissues (**Figure** **9**). Transforming growth factor‐β (TGF‐β) is recognized as a key regulator of skin wound repair, and plays pleiotropic roles at different stages of wound healing by regulating inflammatory responses, fibroblast activation and proliferation, extracellular matrix synthesis, immune responses, and scar formation. Although TGF‐β expression is essential during the inflammatory phase, excessive TGF‐β activity sometimes leads to pathological scar formation.^[^ ^51^ ^]^ α‐Smooth muscle actin (α‐SMA) is one of the markers of fibroblasts transformation into myofibroblasts^[^ ^52^ ^]^ and is crucial in the development and tightening of scar tissue. Induced nitric oxide synthase (iNOS) produces NO in inflammatory responses and has antibacterial and inflammatory effects. Excessive or inappropriate iNOS expression may lead to an increase in inflammatory responses,^[^ ^53^ ^]^ which in turn affects scar formation. This study targeted the late stage of wound healing, and the results showed that the expression of TGF‐β, α‐SMA, and iNOS in the wound of the QOSP hydrogel treatment group was low, while that of the blank group was high, as can be seen from the brighter red fluorescence (Figure 9c). To further investigate the effect of QOSP hydrogel on angiogenesis during the wound healing process, immunofluorescence staining for CD31, a marker for endothelial cells, was performed on day 10 post‐treatment. Figure S15 (Supporting Information) demonstrated that the group treated with QOSP hydrogel exhibited notably lower levels of CD31 expression compared to the blank group, suggesting less angiogenesis at this stage of healing. Quantitative analysis (Figure S16, Supporting Information) further confirmed that the relative area percentage of CD31 was lowest in the QOSP group. This reduction in angiogenesis may be indicative of a shift from tissue regeneration to tissue stabilization, as fewer new blood vessels are needed in the later stages of healing. The data suggest that QOSP hydrogel may support a more controlled transition from angiogenesis to tissue remodeling, promoting balanced healing without excessive vascularization.

These combined effects suggest that QOSP treatment may, through a variety of molecular pathways, reduce inflammation and prohibit excessive fibrosis following burns, enhance the wound's transition from the inflammatory to the proliferative phases, and hasten the healing process. This discovery offers theoretical justification for additional clinical application research as well as molecular‐level evidence in favor of using QOSP to treat burns.

### 2.6 Potential of QOSP for Sensing Applications

**Figure 10:**
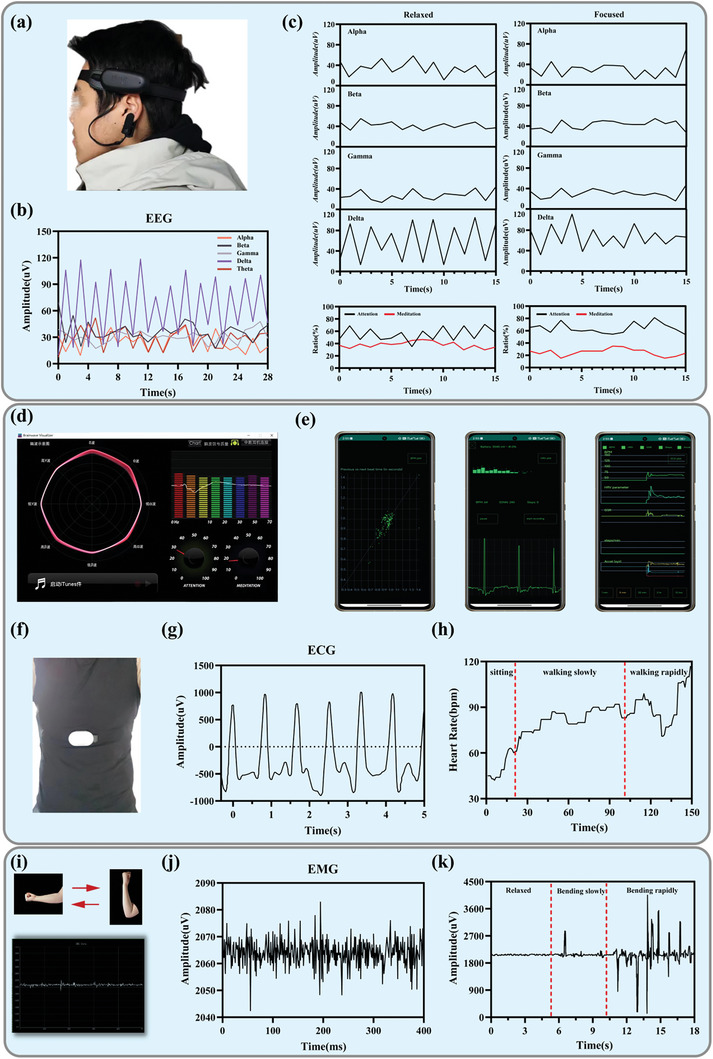
a) Schematic diagram of EEG measurement. b) Waveforms of five types of brain waves in a resting state. c) The four types of brain waves in the states of watching a movie (relaxed) and reading a book (focused), as well as the concentration and relaxation levels of the two states. d,e) EEG sensor host computer interface and ECG sensor mobile terminal interface (HPV heart rate variability analysis image, main interface ECG PLOT and overall data display respectively). f) Schematic diagram of EEG measurement. g) Recorded ECG signals at rest. h) The average heart rate per minute obtained from the ECG signal. The object first sat still, then walked slowly, then walked fast. i) Schematic diagram of arm muscle relaxation and contraction status monitoring and the raw data obtained from the host computer interface. j) Recorded EMG signals of uniform contraction and relaxation. k) Recorded EMG signals when the subject's arm was stationary, contracted slowly, and contracted quickly (Movies 4 and 5, Supporting Information).

Integrating QOSP hydrogels with modern physiological sensors offers a groundbreaking avenue for enhancing the functionality and application range of both materials and devices in biomedical fields.^[^ ^54^ ^]^ QOSP hydrogels, known for their electrical properties and biocompatibility, can be tailored to work in conjunction with various sensors, such as Electroencephalogram (EEG), Electrocardiogram (ECG), and Electromyogram (EMG) devices, to provide comprehensive health monitoring systems. **Figure** **10**a (Supporting Information) presents a schematic of the EEG measurement setup, demonstrating how EEG sensors are positioned on a subject's head to record electrical activity of the brain. This setup is critical for ensuring accurate readings of different brain wave patterns, such as those depicted in Figure 10b, which shows the waveforms of five types of brain waves in a resting state. This data is essential for understanding brain function under various cognitive states. Figure 10c extends this analysis by comparing brain wave patterns during different mental states – relaxed (watching a movie) and focused (reading a book). This comparison is not only crucial for psychological studies, but also for developing applications that enhance cognitive function or monitor mental health. The associated graphs of attention and meditation levels further provide insights into the subject's cognitive load and stress levels, which are valuable for both clinical diagnostics and in designing interfaces that adapt to the user's mental state.

**Figure 11:**
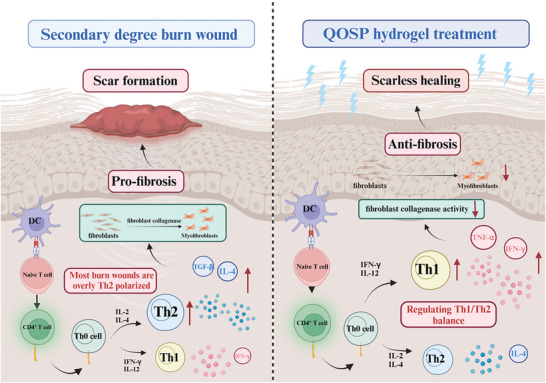
Mechanism diagram of QOSP hydrogel regulating Th1/Th2 balance.

Figure 10d,e demonstrates the interfaces of EEG and ECG monitoring systems, highlighting the advanced technology used to process and display these biological signals. Such interfaces are key to making the data accessible and interpretable by health professionals or patients themselves in real‐time monitoring. Figure 10f represents another EEG setup, underscoring the versatility and adaptability of EEG measurements in various experimental conditions. Meanwhile, image Figure 10g captures an ECG signal at rest, offering a snapshot of cardiac health which is vital for diagnosing heart conditions. The progression of heart rates shown in Figure 10h from rest to active states provides a clear, dynamic view of cardiovascular response during different physical activities, which is crucial for fitness assessments and cardiac rehabilitation. Figure 10i–k focuses on the EMG data. These images highlight how muscle activity can be monitored, showing raw signal outputs and varying intensities of muscle contractions. EMG is instrumental in rehabilitation, sports science, and even in controlling robotic prosthetics or other assistive technologies. As shown in Figure S20 (Supporting Information), the gyroscope‐controlled car can turn based on the arm movements captured by the gyroscope, and changes in muscle electrical signals enable direction changes, highlighting the sensitivity and responsiveness of the system. Furthermore, the EMG waveform diagrams captured by the host computer interface are displayed in Figure S21 (Supporting Information), illustrating the clear signal patterns generated by the armband. Additional serial port debugging data, including EMC and IMU data, are shown in Figure S22 (Supporting Information), confirming the precision and reliability of the system for muscle signal detection and device control. Ultimately, the versatile and adaptable nature of QOSP hydrogels positions them as a critical component in the next generation of medical diagnostics and therapeutic devices, paving new pathways for patient care and health monitoring advancements (**Figure** **11**).

## 3 Conclusion

In this study, we developed an electret‐inspired charge‐injected hydrogel (QOSP hydrogel) designed to promote scar‐free healing of bacterially infected burns through bioelectrical stimulation and immune modulation. The QOSP hydrogel demonstrated significant antibacterial activity, reducing bacterial load by over 90% for SA and PA, and accelerated wound healing by 32% compared to controls. Through its unique ability to store and release electric charges, the hydrogel continuously provided bioelectrical signals to the wound site, enhancing cell migration, proliferation, and differentiation. Moreover, the QOSP hydrogel effectively regulated the immune response by shifting the Th1/Th2 balance toward a Th1‐dominant environment, which is crucial for reducing fibrosis and promoting scar‐free healing. Proteomic analysis revealed down‐regulation of pro‐inflammatory and pro‐fibrotic pathways, further supporting its therapeutic potential. Biodegradability and long‐term charge storage ensure clinical application.

Despite these promising results, several challenges remain. The long‐term stability of the hydrogel under clinical conditions needs to be further evaluated, especially in terms of charge retention and sustained antibacterial efficacy. Moreover, while the animal model results are encouraging, additional studies in larger animals and eventually human clinical trials are necessary to fully understand the safety, biocompatibility, and efficacy of the QOSP hydrogel in diverse patient populations. Another limitation is the potential variability in the electric field distribution across irregular wound surfaces, which could affect the uniformity of bioelectrical stimulation. Future work will focus on optimizing the hydrogel's properties, such as improving charge uniformity, enhancing scalability, and ensuring long‐term biocompatibility in a clinical setting.

In conclusion, this research highlights the potential of bioelectrically active hydrogels as a novel and effective treatment strategy for complex wounds. The QOSP hydrogel offers a promising approach that combines antibacterial activity, immune modulation, and bioelectrical stimulation to accelerate healing and reduce scarring, positioning it as a strong candidate for future clinical wound care applications.

## 4 Experimental Section

### QOSP Fabrication and Synthesis

All reagents and solvents used in this study were of high‐quality reagent grade. Chitosan and glycidyl trimethylammonium chloride (GTMAC) were procured from J&K. Dextran was obtained from Macklin, while APS, SP, and SDI were acquired from Aladdin. Hydrochloric acid (HCl, 36%) and aniline were supplied by Sinopharm Chemical Reagent Co., Ltd. Polystyrene was from Thermo Fisher Scientific and Polystyrene (PS) particles were sourced from Hangzhou Otto Experimental Consumables Co., Ltd. Deionized water was used for all experimental procedures. All reagents were used without further treatment.

The method used to synthesize QCS and OD followed a technique previously described in the literature and polyaniline was synthesized by in situ polymerization (Figure S1, Supporting Information),^[^ ^28^, ^55^ ^]^ which can be found in the Supporting Information. Initially, QCS was dissolved in deionized water to form a QCS solution. Subsequently, PANI and PS were added to this solution, followed by ultrasonic dispersion for 1 h, resulting in a QCS/PANI/PS solution. Separately, OD and SDI were each dissolved in deionized water to create separate solutions of OD and SDI. These solutions, along with the QCS/PANI/PS solution, were combined in various ratios and vigorously shaken to form a conductive hydrogel that facilitates swift SDI release, which was designated as QOSP following plasma treatment. Following this, the hydrogel underwent high‐voltage plasma charge injection to enhance its electrical properties, improving charge storage and conductivity for effective bioelectrical stimulation, named QOSP. Additionally, a hydrogel devoid of plasma charge injection was termed QOS, and a hydrogel comprising only QCS and OD was designated as QO. The components of these hydrogels are detailed in **Table** **1**.

**Table 1:** Concentration (mg/mL) of each component in the hydrogel.

hydrogel	QCS	OD	SDI	PS	PANI
QO	20	35	0	0	0
QOS	20	35	13	10	6
QOSP	20	35	13	10	6

With the discharge electrode acting as the source and the periphery as the ground, the hydrogel sample placed on the tray is exposed to the plasma environment. Under these conditions, positive ions from the plasma are attracted toward the surface of the hydrogel, where they can be deposited, injecting positive charges into the material. Furthermore, the material's components, such as quaternized chitosan (QCS), which contains cationic groups, can help stabilize the injected positive charges. The conductive polyaniline (PANI) nanowires within the hydrogel also facilitate the transfer and retention of these positive charges, enhancing the material's ability to store and maintain the injected charge.

### Characterization of QOSP

The morphology of the QOSP hydrogel and PANI before and after charge injection was investigated using SEM (Regulus 8230, Hitachi Tokyo, Japan) and transmission electron microscopy (TEM: Talos F200x).

To evaluate the thermogravimetric performance of QO, QOS, and QOSP hydrogels, the TGA tests were performed with a TGA/SDTA 851 (Mettler Toledo, Switzerland) at a temperature ramping rate of 10 °C min^−1^. The DSC tests were performed with a DSC 822^e^‐700 (Mettler Toledo, Switzerland) at temperatures ranging from 20 to 800 °C with a ramping rate of 10 °C min^−1^.

A Fourier transform infrared spectroscope (FTIR: Nicolet iS10) was used to determine the FTIR spectrum of the QOSP hydrogel before and after charge injection. The XRD pattern of the QOSP hydrogel was recorded using an XRD spectrometer (ASENWARE, AW‐XDM300, China) over a range of 10 to 80° with a scanning speed of 5°/min. The chemical structure changes in the QOSP hydrogel before and after charge injection were analyzed using XPS. Origin Pro software was used to analyze the results.

The mapping of the surface potential was obtained by KPFM Asylum Research Cypher. The KPFM was operated in non‐contact mode to avoid any potential damage to the sample surface. The instrument was calibrated using a reference material with a known work function before each measurement session. Electrical breakdown characteristics of QOSP‐0 and QOSP‐5 were performed using Keithley 2400 capable of generating voltages up to 210 V. The QOSP samples were sandwiched between two electrodes and a controlled voltage ramp was applied to the samples at a rate of 2 V s⁻^1^ until electrical breakdown occurred. The four‐probe method was employed to measure the electrical conductivity of the QOSP‐0 and QOSP‐5 hydrogels.

The electrical conductivity (σ) of the QOSP‐0 and QOSP‐5 hydrogels was measured using the four‐probe method. The hydrogel samples were prepared in sheet forms to ensure even distribution of current and voltage during measurements. Four metal probes with equal spacing were used as electrodes, with the outer two applying the current and the inner two measuring the voltage. A precision current source provided a constant current to the outer electrodes, while a high‐precision voltmeter measured the voltage drop between the inner electrodes. Multiple measurements were taken and averaged to ensure accuracy. Then the resistivity can be calculated by the Equation (1):

(1) $$\rho =\frac{\pi }{\ln\left(2\right)}\cdot \frac{V}{I}\cdot t$$

where *V* denotes the measured voltage, *I* indicates the applied current and *t* is the thickness of the hydrogel samples. Thus, the conductivity was determined using the Equation (2):

(2) $$\sigma =\frac{1}{\rho }=\frac{\ln\left(2\right)}{\pi }\cdot \frac{I}{V}\cdot \frac{1}{t}$$

Error analysis was performed considering factors such as temperature and contact resistance, with appropriate corrections applied to improve measurement accuracy.

The in vitro degradation of the QOSP hydrogels was assessed by incubating pre‐weighed hydrogel samples in PBS at pH 7.4 and pH 5.5 at 37 °C. At predetermined time intervals (1, 3, 7, 14, 21, and 28 days), the hydrogels were removed, gently washed with distilled water, lyophilized, and weighed. The percentage of residual weight was calculated using the Equation (3):

(3) $$\mathrm{Residual}\;\mathrm{Weight}\;\left(\%\right)=\left(\mathrm{Wt}/W_{0}\right)\times 100\%$$

where Wt is the weight of the hydrogel at time t and W_0_ is the initial weight.

### Coupled Plasma Etching Charge Injection Process

The charge injection into the hydrogel was performed using an instrument set up by the laboratory, and its mechanism of action will be described in detail in the supporting information. The plasma generator selected an aluminum alloy sealed low dielectric vacuum discharge chamber to approximate the total reflection integrating sphere, which is consistent with previous literature reports. The synthesized hydrogel composite was first exposed to ultraviolet light (365 nm, 300 mW cm^−2^) for 5 min using an ultraviolet lamp (Lumen Dynamics Group Inc., Canada). Then, the sample was placed in a capacitively coupled plasma generator (Shaanxi Puguang Microvision Technology Co., Ltd.) and the vacuum pump was evacuated to ≈3 Pa. Then, oxygen entered the vacuum discharge chamber and plasma was generated by capacitively coupled discharge (5 kV) to inject charge into the hydrogel. The samples were classified according to the treatment time: untreated (QOSP‐0), plasma treated for 5 min (QOSP‐5), and plasma treated for 10 minutes (QOSP‐10).

### Biocompatibility Evaluation of QOSP

The biocompatibility of QOSP hydrogels was evaluated by cytocompatibility test and hemocompatibility test. For the cytocompatibility test, a leaching pattern assay was used following the same protocol as in the reference with minor modification.^[^ ^16^ ^]^ Briefly, the sterilized QOSP hydrogels were immersed in the medium for 24h at 37 °C with a shaking speed of 100 rpm to obtain the extract solutions. NIH/3T3 cells were seeded into a 24‐well plate with a cell density of 10000 cells per well, and then replaced the culture medium with hydrogels extract solutions after being pre‐cultured for 24 h. Following the incubation periods of 1, 3, and 7 days, the cells were evaluated. After incubation, the medium from each well (100 µL) was transferred to a new 96‐well plate, and DMEM containing 10% CCK‐8 reagent was added. The absorbance was then measured at 450 nm using a Multiscan microplate reader (Thermo, USA). After being cultured for 7 days, the cell viability under the hydrogels was tested by LIVE/DEAD^®^ Viability/Cytotoxicity Kit assay. For the hemocompatibility test, erythrocytes were isolated from mouse blood through a centrifugation process at 116 ×g for 10 min. The erythrocytes were washed three times with DPBS and diluted to 5% (v/v). Then, 750 uL of erythrocytes were mixed with 750 µL QOSP hydrogel in a 24‐well culture plate, incubated at 37 °C for 1 h with shaking at 100 rpm. The contents were centrifuged again (at 116 ×g, 10 min) to remove hemolysis‐free erythrocytes. 100 µL of supernatant was transferred to a 96‐well plate, and absorbance at 540 nm was measured. Triton X‐100 at a concentration of 0.1% acted as the positive control, while DPBS was the negative control. The hemolysis rate (H) was calculated according to the following Equation (4):

(4) $$H=\frac{Ap-Ab}{At-Ab}\times 100\%$$

where *A_p_* represents the absorbance of the test QOSP hydrogel, *A_t_* represents the absorbance of the positive control (Triton X‐100), and *A_b_* represents the absorbance of the negative control (DPBS).

### Antibacterial Evaluation of QOSP

The antibacterial activity of QOSP hydrogels was assessed using a colony count assay. Gram‐positive *Staphylococcus aureus* (SA) and Gram‐negative *Pseudomonas aeruginosa* (PA) were selected. Briefly, SA and PA (500 µL of 1 × 10^7^ CFU mL⁻^1^) were co‐cultured with QOSP hydrogel samples (500 µL) in a 24‐well plate at 37 °C under aerobic conditions in LB medium for 12 h. The culture medium was then serially diluted and seeded on LB agar plates, followed by an additional 12‐h incubation. The plates were imaged, and the antibacterial efficacy of each hydrogel sample was assessed by tallying the colonies that developed.

### Establishment of Deep Second‐Degree Burn Model

Recent research has found that burns cause more handicaps in women than in males.^[^ ^56^ ^]^ Scars are also more likely to induce depression in women, and treating burn scars in women can help alleviate these issues,^[^ ^57^ ^]^ thus we picked female Kunming mice (30–35g) and made certain alterations based on previous literature studies.^[^ ^16^ ^]^ After anesthesia, the mice's dorsal area was shaved. To induce uniform second‐degree burns, an aluminum rod 1 cm in diameter was heated to 180 °C and pressed vertically against the exposed skin on each mouse's back for 10 s. The wound was round, ≈1 cm in diameter. One hour after the burn, the necrotic tissue was removed with a biopsy punch of the same diameter, similar to the clinical approach for excising badly burned skin. Next, 50 µL of a mixed bacterial solution (50% SA and 50% PA, both at a concentration of 1 × 10^7^ CFU mL⁻^1^) was applied to the wound. After one day, the wound became infected with SA and PA, resulting in the bacteria‐infected deep second‐degree burn model. Then, four kinds of wound dressings were prepared: a) Tegaderm™ film dressing (Blank), b) QO hydrogel, c) QOS hydrogel, d) Charge‐injected QOSP hydrogel. The wound area was imaged at 4, 7, 10, and 13 days of treatment. At the conclusion of the experiment, the mice were euthanized using CO2, and tissue samples were collected for further analysis. Ethical approvals: Experimental Animal Ethics Committee of Zhejiang Chinese Medicine University (No.IACUC‐20221107‐12).

### Histological Analysis

The wound tissues were fixed, dried, paraffin‐embedded, and sectioned using the same process as described in the literature.^[^ ^58^ ^]^ Tissue samples were stained using H&E and the Masson kit (Solarbio Science & Technology, Beijing, China).

### Quantitative Label‐Free Global Proteomic Analysis

Tissue protein extraction was carried out using modified published procedures.^[^ ^59^ ^]^ In brief, 100 mg of protein was extracted from wound tissues treated with Tegaderm™ membrane (Blank) and QOSP hydrogel. Tissues were homogenized and then centrifuged at 10000 ×g for 5 min. The supernatant containing the protein extract was collected and its concentration was measured using the BCA method. Proteins were denatured at 100 °C for 15 min. A 100 µg aliquot of protein sample was diluted in 200 µL of 50 mM ammonium bicarbonate. Disulfide bonds were reduced with 5 mM dithiothreitol (DTT) at 37 °C for 2.5 h, then alkylated with 500 mM iodoacetamide (IAA) for 40 min in the dark. Trypsin (1 µg µL⁻^1^) was used to digest the proteins for 16 h at 37 °C.

To perform a nano‐flow LC‐MS/MS analysis on digested peptides, the samples were made ten times less concentrated using an aqueous solution of 0.1% formic acid from Sigma‐Aldrich. The calculated peptide concentration is 0.2 µg µL^−1^. The peptide sample analysis technique was comparable to that used by Hu and colleagues.^[^ ^60^ ^]^

The protein intensities of the blank group and QOSP hydrogel samples were extracted to indicate their relative levels of expression. Proteins that lacked more than 50% of their label‐free quantification (LFQ) intensity across all samples were excluded. Missing values were generated using a median normalization and k‐nearest neighbors (KNN) imputation. Proteins with intensity coefficients of variation greater than 0.3 were filtered for more accurate quantification results. DEPs between the QOSP groups was assessed using an unpaired Student's t‐test. To account for multiple comparisons, the *p*‐values were adjusted using the Benjamini‐Hochberg (BH) method, with a significance level set at an adjusted *p*‐value of less than 0.05. Proteins exhibiting a fold change greater than 2.0 were classified as significantly up‐regulated or down‐regulated. The biological significance of the identified DEPs was assessed using Gene Ontology (GO) enrichment and Significantly down‐regulated proteins were analyzed in depth by KEGG and Reactome pathways. In addition, PPI network analysis was performed on significantly up‐regulated and down‐regulated differential proteins. The Wu Kong platform (https://www.omicsolution.org/wkomics/main/) was used for statistical analysis and figures were plotted using ggplot2 (https://ggplot2.tidyverse.org/).

### Immunofluorescence Staining

Tissue slices were stained with primary antibodies (anti‐T‐bet, anti‐CXCR3, anti‐GATA3, anti‐TGF‐β1, anti‐α‐SMA, and anti‐iNOS) acquired from Proteintech in Wuhan, China. After a detailed washing, the sections were incubated with secondary antibodies conjugated with FITC (for green fluorescence) or TRITC (for red fluorescence) to enable color detection. The nuclei were then stained using a mounting medium that included 4′,6‐diamidino‐2‐phenylindole (DAPI). The prepared slides were subsequently analyzed using an IX53 inverted fluorescent microscope from Olympus.

### Real‐Time Quantitative Polymerase Chain Reaction (RT‐qPCR)

Jurkat E6‐1 cells, obtained from Suzhou Haixing Biotechnology Co., Ltd., were cultured in RPMI‐1640 medium (C11875500BT, Gibco, Grand Island, NY, USA) supplemented with 10% fetal bovine serum (FBS) and 1% Penicillin‐Streptomycin (15 140 122, Gibco, Grand Island, NY, USA). For activation, we followed the method described in the previous literature and made some minor modifications.^[^ ^61^ ^]^ Jurkat cells (5 × 10^4^ cells per 200 µL per well) were plated in flat‐bottom 96‐well culture plates. These cells were preincubated with anti‐CD3 (10 µg mL⁻^1^ each) for 12 h. Then, activated Jurkat cells are induced to the Th1 and Th2 directions respectively. Th1 conditions: IL‐12 (20 ng mL⁻^1^), IL‐2 (20 ng mL⁻^1^), anti‐IL‐4 (5 ng mL⁻^1^); Th2 conditions: IL‐4 (20 ng mL⁻^1^), IL‐2 (20 ng mL⁻^1^), anti‐IFN‐γ (5 ng mL⁻^1^), all purchased from Proteintech, Wuhan, China. After 12 h of induction, the Th2‐directed cells were co‐cultured with QO, QOS, and QOSP hydrogels for 24 h. After the culture was completed, the cells were harvested and RNA was extracted using the Trizol Reagent (Life Technologies) technique. RNA was reverse transcribed into cDNA using the PrimeScript™ RT reagent kit (Takara) procedure. RT‐qPCR was used to measure relative mRNA expression levels using the LightCycler® 96 instrument (Roche Diagnostics, USA) with TB Green® Premix Ex Taq™ II (Takara, Japan). The primer sequences (obtained from Shanghai Shenggong Biotech Co., Ltd) for RT‐qPCR are listed below: CXCR3 (Fwd: CCACCTAGCTGTAGCAGACAC, Rev: AGGGCTCCTGCGTAGAAGTT), IFN‐γ (Fwd: TCGGTAACTGACTTGAATGTCCA, Rev: TCGCTTCCCTGTTTTAGCTGC), GATA3 (Fwd: GCCCCTCATTAAGCCCAAG, Rev: TTGTGGTGGTCTGACAGTTCG), IL‐4 (Fwd: CCAACTGCTTCCCCCTCTG, Rev: TCTGTTACGGTCAACTCGGTG).

### Western Blotting

The expression of protein level was measured by western blotting technology. Proteins underwent separation using SDS‐PAGE in a 12% gel and were subsequently transferred to a PVDF membrane provided by Millipore (IPVH00010). The membrane was blocked for 1 h at room temperature using 5% non‐fat dry milk in TBST buffer (20 mM Tris, 150 mM NaCl, 0.1% Tween‐20, pH 7.5). Subsequently, the membrane was incubated overnight at 4 °C with primary antibodies directed against TLR3 (Toll‐like receptor 3, 17766‐1‐AP, ProteinTech, 1:1000), JAK3 (Janus kinase 3, 80331‐1‐RR, ProteinTech, 1:1000), STAT2 (Signal transducer and activator of transcription 2, 16674‐1‐AP, ProteinTech, 1:1000), MLKL (Mixed lineage kinase domain‐like protein, 66675‐1‐Ig, ProteinTech, 1:1000), TAGLN2 (Transgelin 2, 10234‐2‐AP, ProteinTech, 1:1000), JAK2 (Janus kinase 2, R24775, Zen‐Bioscience), Phospho‐JAK2 (Janus Activating Kinase 2, 680 621, Zen‐Bioscience), STAT4 (Signal transducer and activator of transcription 4, R27319, Zen‐Bioscience), Phospho‐STAT4 (Signal transducer and activator of transcription 4, ab313630, Abcam plc) and GAPDH (60004‐1‐Ig, ProteinTech, 1:5000) as loading control. After washing thrice with TBST, the membrane was incubated with horseradish peroxidase‐conjugated secondary antibody (Goat anti‐rabbit IgG, 1:5000, Sigma‐Aldrich; Goat anti‐mouse IgG, 1:5000, Sigma‐Aldrich) for 1 h at room temperature. Chemiluminescent detection was performed using the ECL Western Blotting Detection System (Amersham Biosciences).

### Flow Cytometry

Jurkat cells were cultured in RPMI‐1640 medium supplemented with 10% fetal bovine serum (FBS), 1% penicillin/streptomycin, and 2 mM L‐glutamine at 37 °C in an incubator with 5% carbon dioxide. CD3 antibody was used to activate cells at a concentration of 10 µg mL⁻^1^ for 12 h. The control group was set up without any material. The quaternized chitosan, QO, and QOSP hydrogels were co‐cultured with Jurkat cells at 0.1% (w/v) for 24 h, and then the cells were harvested, washed with PBS, and stained with the following antibodies for phenotypic analysis: APC anti‐human CD183 (CXCR3) Antibody (Catalog number 353707) and PE anti‐human CD194 (CCR4) Antibody (Catalog number 359411) were purchased from Biolegend lnc. Flow cytometric analysis was performed using a CytoFLEX flow cytometer, and the expression of CXCR3 and CCR4 was analyzed using CytExpert v2.6 software, and the percentage of positive cells was determined by comparing the fluorescence intensity with the isotype control. Statistical differences were determined by GraphPad Prism software, and *p* < 0.05 was considered statistically significant.

### Elisa Assay

To quantify the production of IFN‐γ, IL‐2 and IL‐12 in T cells, Jurkat cells (5 × 10^4^ cells per 200 µL per well) were plated in flat‐bottom 96‐well culture plates. These cells were preincubated with anti‐CD3 (10 µg mL⁻^1^ each) for 12 h. After co‐culturing Jurkat cells with quaternized chitosan, QO hydrogel, QOSP‐5 hydrogel for 24 h, the culture medium was collected. The levels of IFN‐γ (catalog number KE00146), IL‐2 (catalog number KE00017), and IL‐12 (catalog number KE00018) were quantified using an ELISA kit from Proteintech (Wuhan, China) according to the manufacturer's instructions.

### Statistical Analysis

All quantitative data were reported as mean ± SD. For properly distributed data sets with equal variances, a one‐way ANOVA with a Tukey post‐hoc test was used across groups. The statistical analysis was carried out using GraphPad Prism Software (GraphPad Prism, CA, USA). Significance was judged as *p* < 0.05. The sample size (n) was at least three.

## Conflict of Interest

The authors declare no conflict of interest.

## Supporting information



Supporting Information

## Data Availability

The data that support the findings of this study are available in the Supporting Information of this article.
