# Supplementary material for: Electret‐Inspired Charge‐Injected Hydrogel for Scar‐Free Healing of Bacterially Infected Burns Through Bioelectrical Stimulation and Immune Modulation
Source: Adv Sci (Weinh). 2025 Feb 14;12(13):2411889. doi: 10.1002/advs.202411889 (PMC11967837; doi:10.1002/advs.202411889)
Supplement: Supplementary file 1 — Supporting Information [file ADVS-12-2411889-s001.docx]

Supporting Information

**Electret-Inspired Charge-Injected Hydrogel for Scar-Free Healing of Bacterially Infected Burns through Bioelectrical Stimulation and Immune Modulation**

*Mujie Liu, Yuheng Wang*, Haodong Wang, Lihong Qi, Yuxuan Shang, Jiajie Song , Xiulong Feng, Yiwei Chen, Waqar Ali Memon, Yuping Shen, Xiaodong Wu, Jiangbei Cao, Yifan Zhao, Zhuangde Jiang, Dingxin Liu, Shareen Shafique, Shengtao Li, Guanghao Lu, Zhixiang Wei , Zhijie Liu, Kun Zhou, Yuping Quan, Xiaoyu Zhang, Xin Zou, Xuefeng Wang, Na Liu, Yaqing Zhang*, Yiwei Hu*, Chao Han*, Wen Wang**

M.Liu, Y.Wang, Y.Shang, J.Song, X.Feng, X.Wang, N.Liu, W.Wang

Functional and Molecular Imaging Key Lab of Shaanxi Province, Department of Radiology, Tangdu Hospital

Air Force Medical University

Xi’an 710032, China

E-mail: wangwen@fmmu.edu.cn

nano.plasma.uv@gmail.com

M.Liu, H.Wang

Health Science Center

Ningbo University

Ningbo 315211, China

Y.Wang, D.Liu, S. Li, G.Lu, Z.Liu

State Key Laboratory of Electrical Insulation and Power Equipment, Centre for Plasma Biomedicine, School of Electrical Engineering

Xi’an Jiaotong University

Xi’an 710049, China

Email: nano.plasma.uv@gmail.com

Q.Li

Department of Geriatric Medicine

The Affiliated Hospital of Southwest Medical University

Luzhou 646000, China

Y.Chen, Y.Hu

Department of Orthopedic Surgery

Shanghai Sixth People's Hospital Affiliated to Shanghai Jiao Tong University School of Medicine

Shanghai 200233, China

E-mail: hu.yiwei@shsmu.edu.cn

Waqar Ali Memon

Shenzhen Grubbs Institute and Department of Chemistry

Southern University of Science and Technology

Shenzhen 518055, China

Y.Shen, C.Han

The Second Affiliated Hospital of Zhejiang Chinese Medical University

Hangzhou 310000, China

E-mail: 20224012@zcmu.edu.cn

X.Wu, J.Cao

Department of Anesthesiology

the First Medical Center of Chinese PLA General Hospital

Beijing 100853, China

Y.Zhao, Z.Jiang, Shareen Shafique

State Key Laboratory for Manufacturing Systems Engineering

International Joint Laboratory for Micro/Nano Manufacturing and Measurement Technologies

School of Instrument Science and Technology

Xi’an Jiaotong University

Xi’an 710049, China

Z.Wei

CAS Key Laboratory of Nanosystem and Hierarchical Fabrication

CAS Center for Excellence in NanoscienceNational Center for Nanoscience and Technology

Beijing 100190, China

Z.Kun

School of Science and Engineering

Shenzhen Institute of Aggregate Science and Technology

The Chinese University of Hong Kong, Shenzhen (CUHK-Shenzhen)

Guangdong 518172, China

Y.Quan

Department of Plastic Surgery and Regenerative Medicine

Fujian Medical University Union Hospital

Fuzhou 350001, China

X.Zhang, X.Zou

Department of Medical Engineering, Xinqiao Hospital

Army Medical University

Chongqing 400037, China

Y.Zhang

Department of Pediatric Orthopaedics

Xinhua Hospital Affiliated to Shanghai Jiao Tong University, School of Medicine

Shanghai 200092, China

E-mail: zhangyaqingmed@163.com

**Supplementary Text**

[Supplementary 1: Materials and methods 4](#_Toc1454)

[1.1. Synthesis of quaternized chitosan (QCS) 4](#_Toc32708)

[1.2. Synthesis of oxidized dextran (OD) 4](#_Toc23477)

[1.3. Characterizations 5](#_Toc6644)

[1.4. QOSP-5 or QOSP-10 5](#_Toc5873)

[Supplementary 2: Equipment 6](#_Toc12033)

[Supplementary 3: Thermally stimulated depolarization current (TSDC) 7](#_Toc22311)

[Supplementary 4: Effects of electrical stimulation on cell tropism 7](#_Toc30050)

[Supplementary 5: Immunofluorescence Staining 8](#_Toc24475)

[Supplementary 6: Real-time quantitative Polymerase Chain Reaction (RT-qPCR) 9](#_Toc30568)

[Supplementary 7: Quantitative label-free global proteomic analysis 9](#_Toc32003)

[Supplementary 8: Immunomodulatory effect of quaternized chitosan in QOSP hydrogel 10](#_Toc21463)

[Supplementary Figures: 11](#_Toc27743)

[Supplementary References: 28](#_Toc4078)

# Supplementary 1: Materials and methods

## Synthesis of quaternized chitosan (QCS)

The QCS was synthesized according to a previously published technique.^[1]^ One gram of chitosan was suspended in 36 milliliters of acetic acid solution (0.5% v/v). 1.16 mL of GTMAC was added to the suspension in drops at a time. The reaction mixture was then heated to 55 °C and stirred for 18 hours. Following this, the mixture was centrifuged at 7000 rpm for 15 minutes, and the supernatant was precipitated with acetone. This purification procedure was performed three times to verify the purity of the finished product, which was then dried in a vacuum oven. The amount of quaternization in the QCS was evaluated by titrating the chloride ions present.

## Synthesis of oxidized dextran (OD)

The synthesis of OD follows a previously described protocol.^[2]^ 1 gram of dextran was dissolved in 100 milliliters of deionized water. This was followed by the dropwise addition of 856 mg sodium periodate, which had been dissolved in 8 mL of deionised water. The mixture was then mixed in the dark for four hours at room temperature. To stop the process, 1 mL of ethylene glycol was added. The resultant solution was then extensively dialyzed against deionized water and lyophilized to yield OD.

## Characterizations

The morphology of QOSP hydrogels was observed by a scanning electron microscopy (SEM: Regulus 8230, Hitachi Tokyo, Japan).

**Figure S6** complements the XPS results from QOSP-0, QOSP-5, and QOSP-10. In the C 1s spectrum (**Figure S6a-c**), the untreated sample (QOSP-0) has the primary C-C/C-H peaks at 51.46%, whereas C-O (9.14%) and C=O (5.11%) are less prominent. After 5 minutes of plasma treatment (QOSP-5), the C-O peak jumped quickly to 39.4%, indicating accelerated oxidation. C-C/C-H and C=O adjusted to 20.93% and 21.03% respectively. Extending the treatment duration to 10 minutes (QOSP-10) increased the C-O peak to 43.6%, but the C-C/C-H and C=O peaks declined, indicating ongoing surface alteration. The O 1s spectra (**Figure S6d-f**) demonstrate that -OH groups initially were 35.98% in QOSP-0 and rose dramatically to 63.77% in QOSP-5 and 70.66% in QOSP-10, showing that hydroxyl groups The level of culturalization has greatly improved. The intensities of C=O and C-O adapt to reflect the increased surface oxygen content. Overall, charge injection can strengthen the carbon-oxygen bonds and hydroxyl groups on the hydrogel's surface, improving its oxidative and hydrophilic properties.

## QOSP-5 or QOSP-10

In our final decision to select QOSP-5 for our experiments, we considered both the material performance and practical economic factors. The surface potential of QOSP-5, ranging from 296.3 mV to 363.5 mV, provides sufficient charge storage to produce the necessary bioelectrical stimulation without the need for the higher voltages of QOSP-10, which ranges up to 414.4 mV. Importantly, the conductivity of QOSP-5 (3.33 × 10^-5^ S/m) is already significantly enhanced compared to QOSP-0, and this level of conductivity is more than adequate for the intended wound healing applications.

Mathematically, using the surface potential of 300 mV and assuming an effective electric field interaction range of 1 µm, we can calculate the electric field strength for QOSP-5 as:

E = V / d = (300 × 10^-3^ V) / (1 × 10^-6^ m) = 300 kV/m

With this electric field strength and the conductivity value, we estimate the current density for QOSP-5:

J = σ E = (3.33 × 10^-5^ S/m) × (300 × 10^3^ V/m) = 10 A/m^2^

This current density is more than sufficient to generate bioelectrical stimulation at the wound site to promote cell migration and proliferation.^[3]^ Moreover, while QOSP-10 may provide slightly higher surface potential and conductivity, its increased production time (10 minutes of plasma treatment vs. 5 minutes for QOSP-5) and potentially reduced flexibility make it less practical for scalable applications. Furthermore, the improved mechanical properties and sufficient electrical performance of QOSP-5 make it a more balanced choice, especially for routine wound care applications that do not require the extreme electrical properties offered by QOSP-10. Thus, based on both experimental results and cost-efficiency considerations, QOSP-5 was chosen as the optimal formulation for our ongoing studies.

# Supplementary 2: Equipment

**Figure S10** shows the charge injection device we designed to inject charges into hydrogels. The Capacitive Coupling Plasma (CCP) system generates plasma containing a mix of free electrons, positive ions, and negative ions. Due to mass of electrons compared to ions, electrons tend to escape from the surface more easily, while positive ions (such as argon or oxygen ions) are slower and can accumulate on the surface of the sample, leading to positive charge deposition. The basic structure of an inductively coupled plasma generator includes an RF power supply, a system control section, and a vacuum and gas circuit section. The RF power supply module is designed to provide the precise RF energy required to start and maintain the plasma in the system. Its main RF source is a cylindrical quartz tube wrapped with a high-frequency (13.56 MHz) current coil. While ensuring normal operation at low voltage, it also includes power amplification and regulation, RF regulation, output protection, temperature protection and other parts. The system control unit comprehensively supervises and regulates the operation of the generator to ensure the stability and accuracy of plasma production. The system control section includes digital automatic control, I/O control, vacuum A/D detection, pulse width ratio D/A adjustment, parameter memory, clock setting and other parts. The vacuum and gas circuit manages the delicate balance of gas and maintains the necessary vacuum conditions, which is essential for the effective operation of the plasma generator. The vacuum and gas section includes the vacuum system, applied gas, flow regulation, return gas and pipelines. The ICP-FLAS equipment adopts a dual gas input design, and the gas system can choose to use a single gas or a mixed gas. It is worth noting that the RF power supply adopts a compact design, allowing the RF power supply and system control components to be integrated in a more space-saving way. This not only saves valuable physical space within the instrument, but also simplifies the overall assembly and operating procedures, improving the efficiency and ease of use of the system.

# Supplementary 3: Thermally stimulated depolarization current (TSDC)

As shown in the **Figure S11**, the thermally stimulated depolarization current (TSDC) results of QOSP-0 and QOSP-5 show different thermal responses between the two materials. QOSP-0 exhibits a clear depolarization peak around 90 °C, indicating a sharp release of stored charges at this temperature, which may be due to structural transformation within the hydrogel matrix. In contrast, QOSP-5 shows a smaller and broader depolarization peak around 140 °C, which may indicate an increase in cross-linking density or a more uniform charge distribution, thereby enhancing the thermal stability of the hydrogel. This is consistent with previous analysis, indicating that components such as polyaniline and polystyrene integrated into QOSP-5 contribute to improved thermal properties. In addition to this, the shift and decrease in peak intensity in QOSP-5 indicate a change in electrical functionality due to the charge injection process. This enhancement makes QOSP-5 potentially more suitable for applications that require stable electrical properties under different thermal conditions. In summary, the TSDC results are consistent with the hypothesis that charge injection can improve the ability of hydrogels to handle thermal and electrical stresses, which confirms the results of other characterizations such as DSC and conductivity testing. This comprehensive approach confirms the potential of QOSP-5 for advanced applications in fields that require robust and reliable materials.

# Supplementary 4: Effects of electrical stimulation on cell tropism

Electrical stimulation can significantly influence the morphology and behavior of cells through several key mechanisms. When an electric field is applied, it induces changes in the cell membrane potential, leading to the activation of various ion channels.^[4]^ The influx of ions, particularly calcium ions (Ca²⁺), triggers intracellular signaling cascades such as those involving protein kinase C (PKC) and the PI3K/AKT phosphatidylinositol-3-kinase/Akt signal transduction pathway. These pathways play crucial roles in regulating cytoskeletal dynamics, including the organization of actin filaments and microtubules, which are essential for cell shape and motility. The electrical field can also affect the secretion of extracellular matrix (ECM) components by the cells, which in turn influences cell adhesion and alignment. Together, these mechanisms contribute to the phenomenon of electrotaxis, where cells migrate or align in response to the direction and intensity of the applied electric field.^[5]^

As shown in **Figure S12**, NIH/3T3 cells were subjected to electrical stimulation at different current intensities (0 mA, 5 mA, 10 mA, and 20 mA) for 2 hours, leading to observable changes in their morphology and alignment. At 0 mA, the cells displayed no significant changes in morphology or alignment, serving as the control group. Upon exposure to 5 mA, slight cellular alignment and elongation were noted, indicating the initiation of an electro-tropic response. This suggests that even a moderate current can start to activate the signaling pathways responsible for cytoskeletal reorganization. At 10 mA, the most pronounced cellular changes were observed. The majority of cells showed clear directional alignment and an elongated morphology, consistent with optimal activation of the aforementioned intracellular pathways. This alignment indicates a strong electro-tropic response, where the cells are responding to the electric field by reorienting themselves, possibly toward the anode.However, at 20 mA, despite some cells maintaining alignment, a significant number of cells began to exhibit signs of cytotoxicity. These signs included cell rounding and detachment from the substrate, which are indicative of cellular stress or death. This suggests that at higher current intensities, the electrical stimulation might overstimulate ion channels, leading to detrimental effects such as excessive calcium influx and activation of apoptotic pathways.

This phenomenon provides valuable insights into the development and application of self-powered charge-injected hydrogels. These hydrogels, when integrated into biomedical devices or tissue engineering scaffolds, can harness the body's own electric fields or generate local fields to guide cell behavior in a controlled manner. The results show that carefully tuning the electrical properties of the hydrogel, such as its conductivity and the strength of the electric field it generates, can optimize cellular responses, such as alignment and proliferation, which are critical for effective tissue regeneration. This highlights the potential of this new class of hydrogels in regenerative medicine, where they can serve not only as structural supports but also as active participants in guiding cell organization and healing processes.

# Supplementary 5: Immunofluorescence Staining

Based on the proteomic findings, we investigated the effect of QOSP hydrogels on angiogenesis in more detail, and we performed immunofluorescence staining for CD31, a well-established endothelial cell marker, to assess the effects of QOSP hydrogels on wound tissue during critical stages of healing (**Figure S15-S16**). The degree of vascularization. CD31 expression was significantly lower in the QOSP group compared with the blank group, indicating reduced angiogenesis. This observation was confirmed quantitatively by the relative area coverage of CD31, which was lowest in the QOSP group. CD31 expression also decreased in the QO and QOS groups, but to a smaller extent, suggesting a smaller effect on angiogenesis. In fact, it is not uncommon for angiogenesis to decrease in the late stages of wound healing. Hu etal. also found that wounds in diabetic mice also had decreased vascularization in the late stages of healing.^[6]^ This is because as the wound enters the later stages of healing and the need for new blood vessel formation gradually decreases, QOSP hydrogel may promote the transition from angiogenesis to a fibrotic environment, where the focus shifts from tissue regeneration to tissue stabilization. At the same time, in the later stages of healing, the late anti-inflammatory effect of QOSP hydrogel further inhibited angiogenesis. These results further confirmed the proteomic conclusions.

# Supplementary 6: Real-time quantitative Polymerase Chain Reaction (RT-qPCR)

We examined the impact of QOSP hydrogel on the control of Th1/Th2 equilibrium in vitro and ascertained the mRNA composition of its manifestation. **Figure 7d-e** and **Figure S17** displays the findings. Th1 cells have a specific target in IFN-γ, and their membrane contains the chemokine receptor CXCR3. Th2 cells have a unique target in IL-4, and one transcription factor that is exclusive to Th2 cells is GATA3. The outcomes demonstrated that Th1 and Th2 cells were successfully produced. Following co-culturing Th2 cells with QO, QOS, and QOSP hydrogels, there was a decrease in GATA3 and IL-4 expression and varied increases in CXCR3 and IFN-γ, which were on par with or even higher than the Th1 positive control group. The findings demonstrate that QOSP hydrogel has the ability to control T cell polarization toward Th1, as well as deftly control the Th1/Th2 balance at the site, which prevents fibrosis and encourages scar-free wound healing.

# Supplementary 7: Quantitative label-free global proteomic analysis

The PCA results for the QOSP and Blank groups are displayed in **Figure S18b**. It is evident that there are notable variations in the expression patterns between the QOSF dressing group and the blank control group, as they are clearly separated on the first principal component (PCA1). **Figures S18c–d** depict the PPI network analysis of up-regulated and down-regulated differentially expressed proteins (DEPs), respectively. It is worth noting that in the down-regulated PPI map, important proteins like as Rpl24, Prkaca, and Myh7 are highlighted as central nodes. Rpl24 is a ribosomal protein that participates in the translation process of protein synthesis; Prkaca is a protein kinase A (PKA) is the catalytic subunit of Myh7 that is activated by cAMP and participates in the signaling of various cellular processes; Myh7 is a myosin heavy chain found primarily in cardiac and skeletal muscles and is responsible for muscle contractility. Down-regulation of these proteins may jointly promote the smooth transition from the inflammatory phase to the proliferative phase, reduce fibrosis in tissues, and inhibit excessive angiogenesis, further corroborating the results of KEGG and Reactome pathway analyses.

# Supplementary 8: Immunomodulatory effect of quaternized chitosan in QOSP hydrogel

To investigate the potential of chitosan, QO and QOSP hydrogels in modulating T cell differentiation, activated Jurkat cells were co-cultured with these materials for 12 h. Flow cytometry analysis (as shown in **Figure S23**)was performed to assess the expression of key chemokine receptors, CXCR3 and CCR4, which are markers of Th1 and Th2 differentiation, respectively. Jurkat cells co-cultured with quaternized chitosan (QCS), QO and QOSP hydrogels exhibited a marked upregulation of CXCR3 expression and a near-complete absence of CCR4 expression, indicating a bias towards Th1 differentiation. It is worth mentioning that the differences between the three groups were not significant. The blank group, consisting of activated Jurkat cells, showed low level of both CXCR3 and CCR4 expression.

During the differentiation process of T cells, the Jak2-Stat4 signaling pathway plays a key role in the formation of Th1 cells. The differentiation of Th1 cells depends on the activation of the Jak2-Stat4 axis mediated by IL-12 and IFN-γ. IL-12 activates JAK2 by binding to its receptor, thereby promoting the phosphorylation of Stat4. Phosphorylated Stat4 is then translocated into the nucleus and initiates transcriptional regulation with Th1-specific transcription factors (such as T-bet) and cytokine genes (such as IFN-γ). The activation of Jak2 and the phosphorylation of Stat4 reflect the role of Th1 cells in immune responses, especially in anti-viral and anti-tumor immunity.^[7–9]^ Through Western blot analysis, a significant increase in the phosphorylation levels of Jak2 and Stat4 was observed in T cells treated with QCS, QO and QOSP hydrogels (**Figure S23**), indicating the functional role of these molecules in cell fate determination. These data support the central role of the Jak2-Stat4 signaling pathway in Th1 differentiation and provide a theoretical basis for its immunotherapy research as a potential target.

The ELISA and PCR results depicted in **Figure S24** further substantiate the previously drawn conclusions, particularly emphasizing cytokine production and gene expression. The findings reveal that T cells co-cultured with QCS, QO and QOSP hydrogels all demonstrated significant Th1 induction. In detail, compared to the Blank group, QCS significantly boosted the production of IFN-γ, IL-2, and IL-12, with concentrations reaching 350 pg/ml, 151 pg/ml, and 89 pg/ml, respectively. Complementary RT-qPCR analysis showed that the relative expression levels of CXCR3 and IFN-γ in the group treated with quaternary ammonium chitosan were elevated by 40 times and 25 times, respectively, compared to the Blank group, with no marked differences observed between the QO and QOSP groups.

These results are aligned with the goals of immune regulation and confirm that the Th1-inducing effect of QOSP hydrogel is primarily attributed to quaternized chitosan. This insight underscores the potential of quaternized chitosan to consistently promote a Th1-type immune response, providing robust support for our research hypothesis.

# Supplementary Figures:


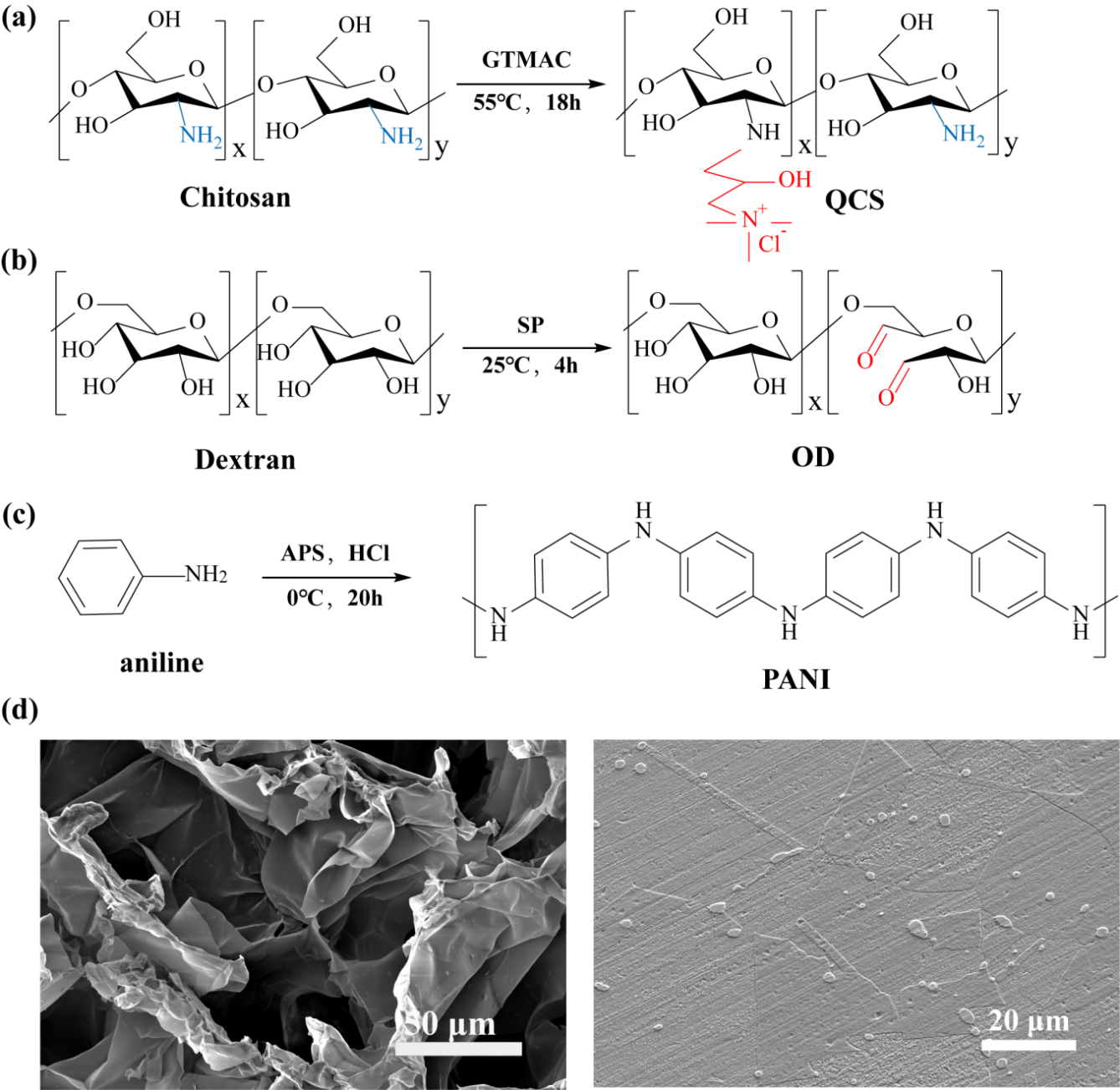


**Figure S1**. Synthetic routes of QCS (a), OD (b) and PANI (c). (d) Surface morphology of QOSP-5 hydrogel observed by SEM.


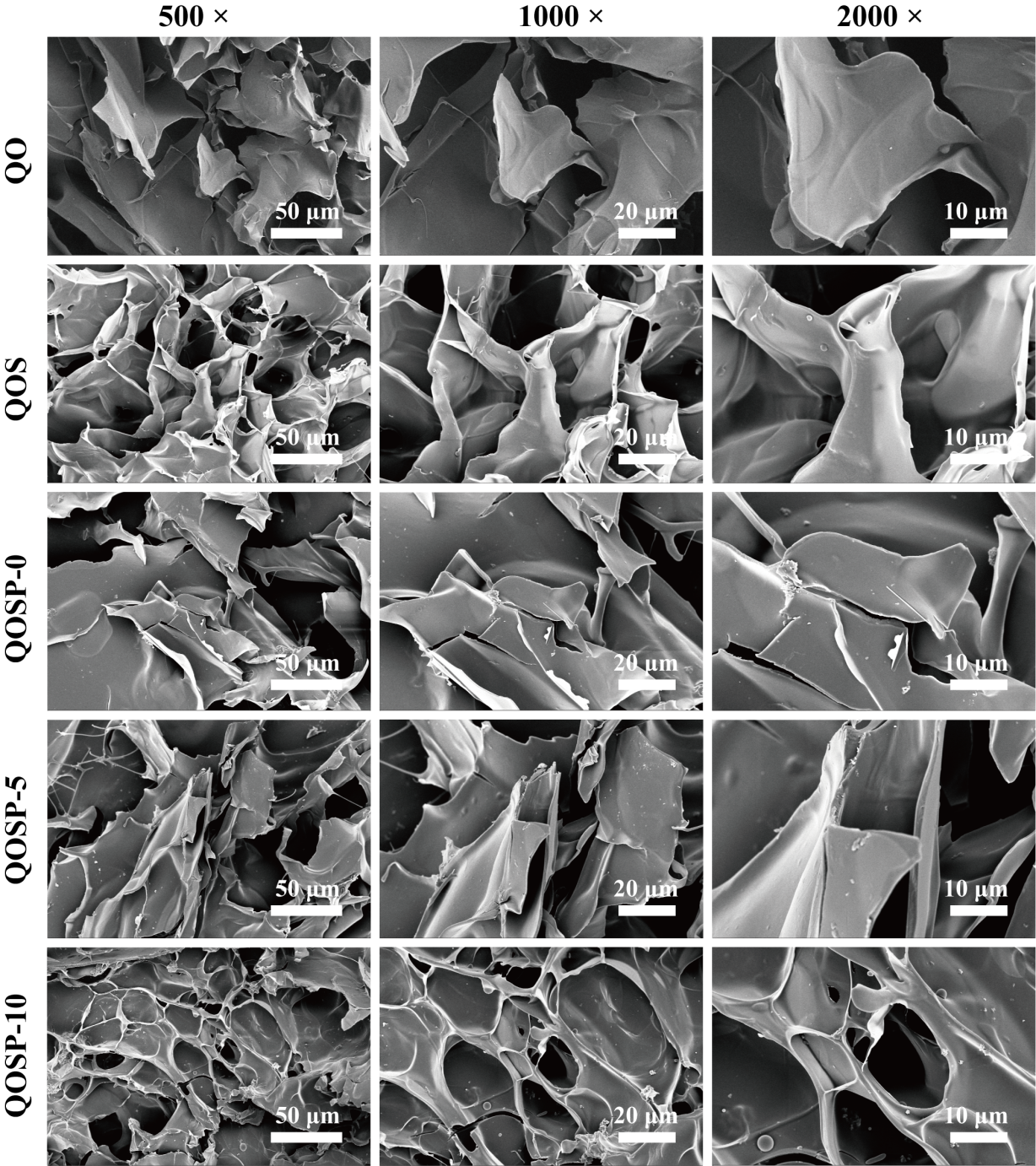


**Figure S2**. SEM images of QO, QOS, QOSP-0, QOSP-5, QOSP-10 hydrogels, magnified at 500×, 1000×, and 2000×, respectively.


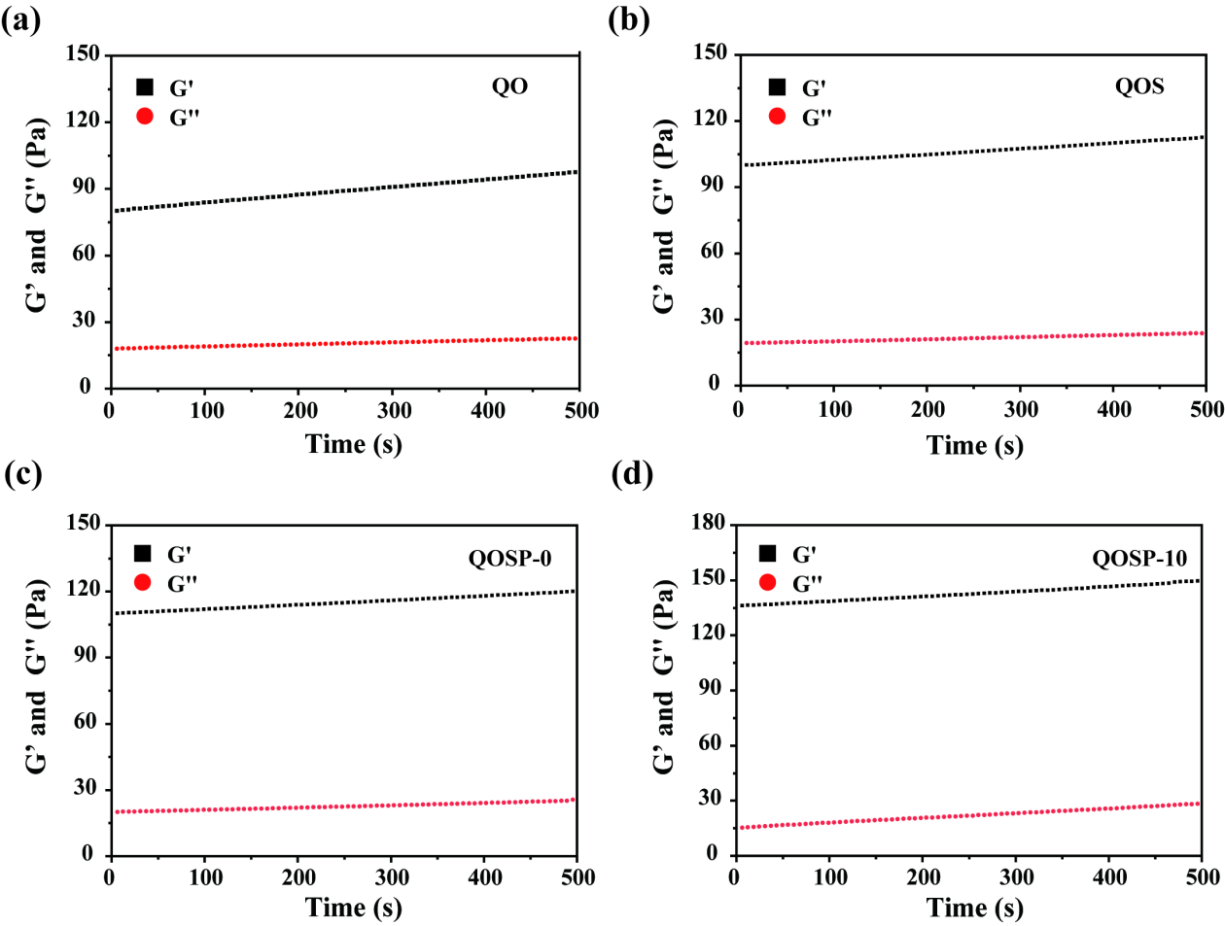


**Figure S3.** Rheological properties of (a) QO, (b) QOS, (c) QOSP-0 and (d) QOSP-10 hydrogels.


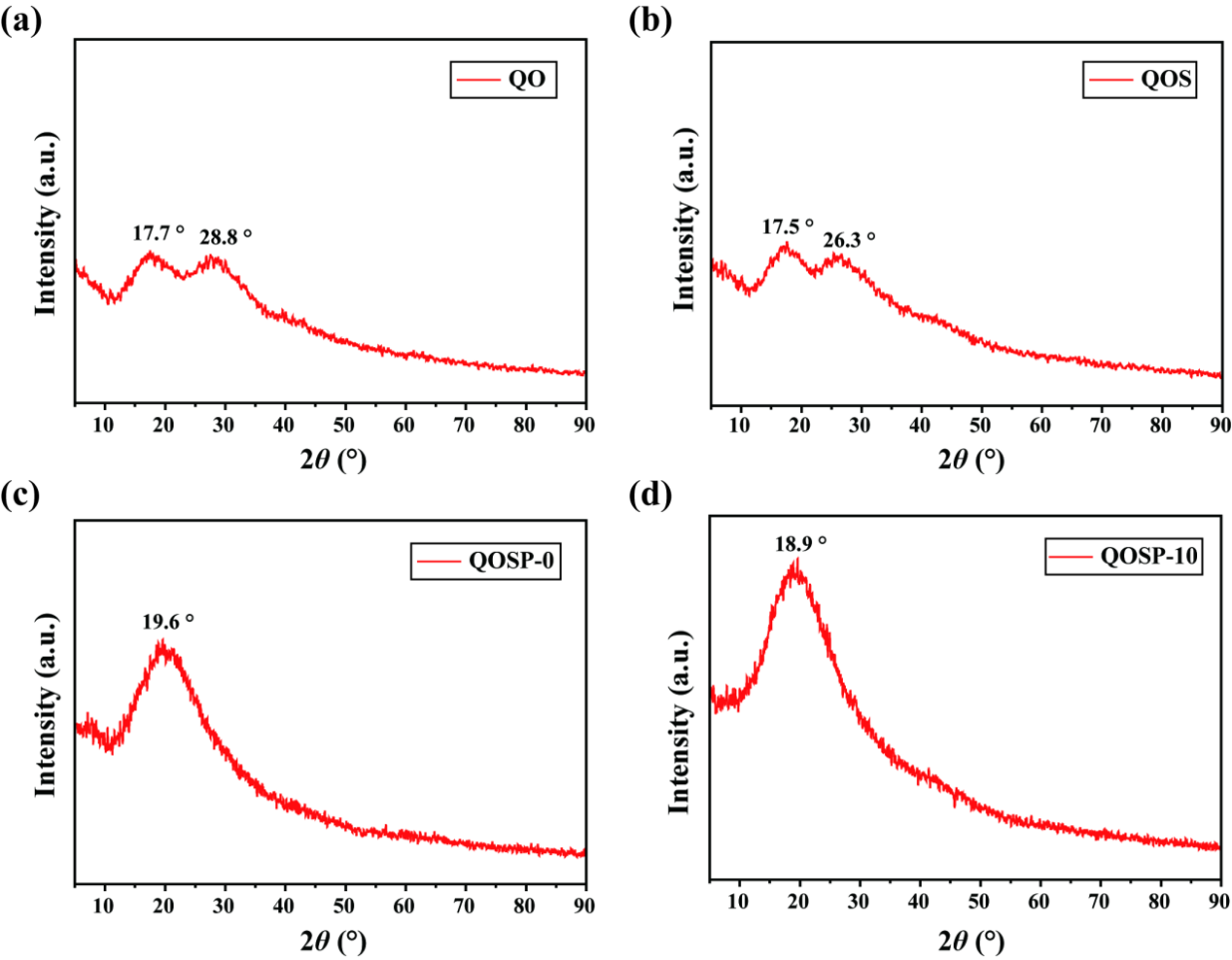


**Figure S4.** XRD analysis of (a) QO, (b) QOS, (c) QOSP-0 and (d) QOSP-10 hydrogels.


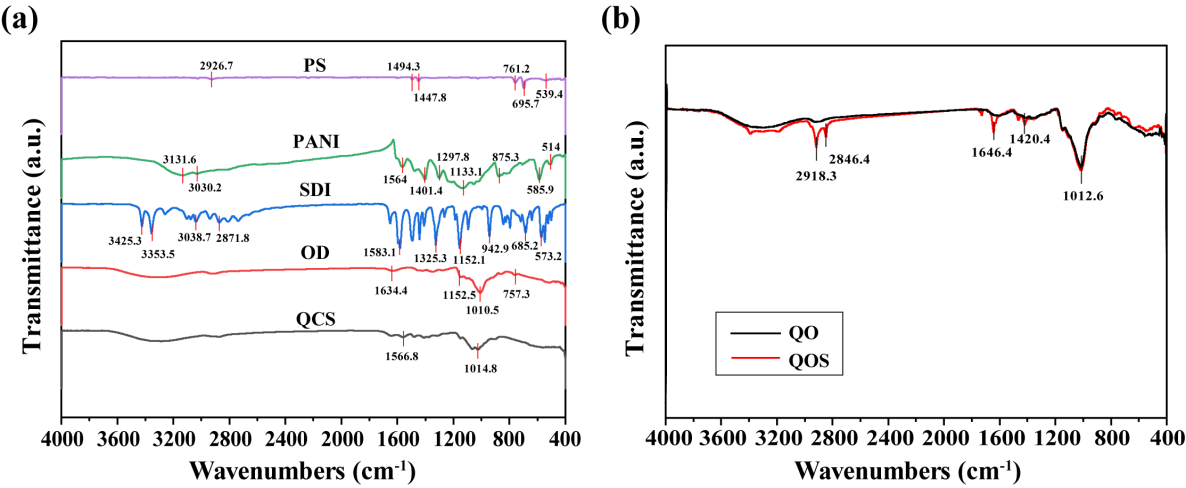


**Figure S5**. (a) FTIR spectra of QCS, OD, SDI, PANI and PS. (b) FTIR spectra of QO and QOS hydrogles.


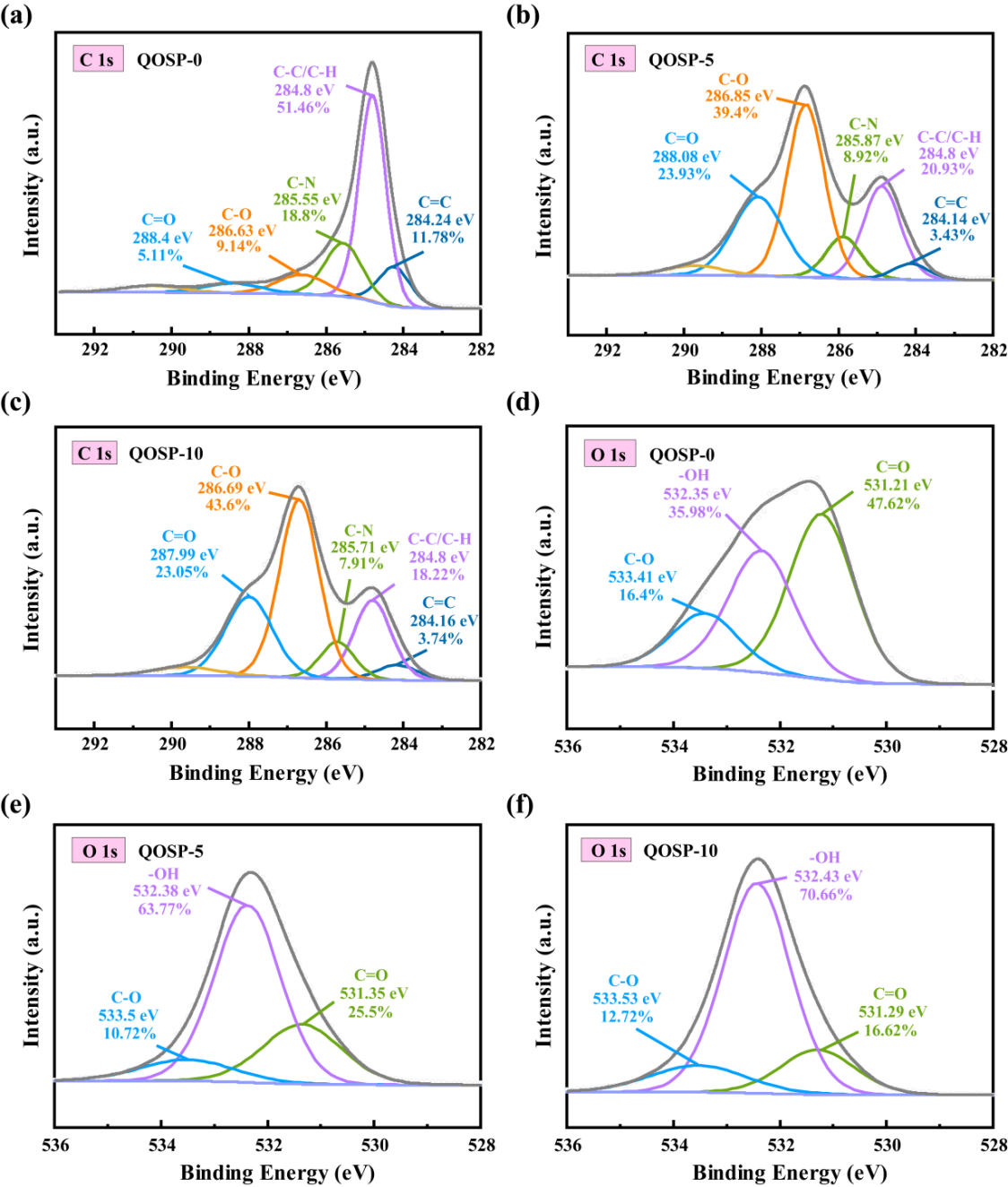


**Figure S6**. (a)-(c) X-ray Photoelectron Spectroscopy (XPS) spectra of C 1s for QOSP-0, QOSP-5 and QOSP-10 hydrogels. (d)-(f) XPS results of O 1s for QOSP-0, QOSP-5 and QOSP-10 hydrogels.


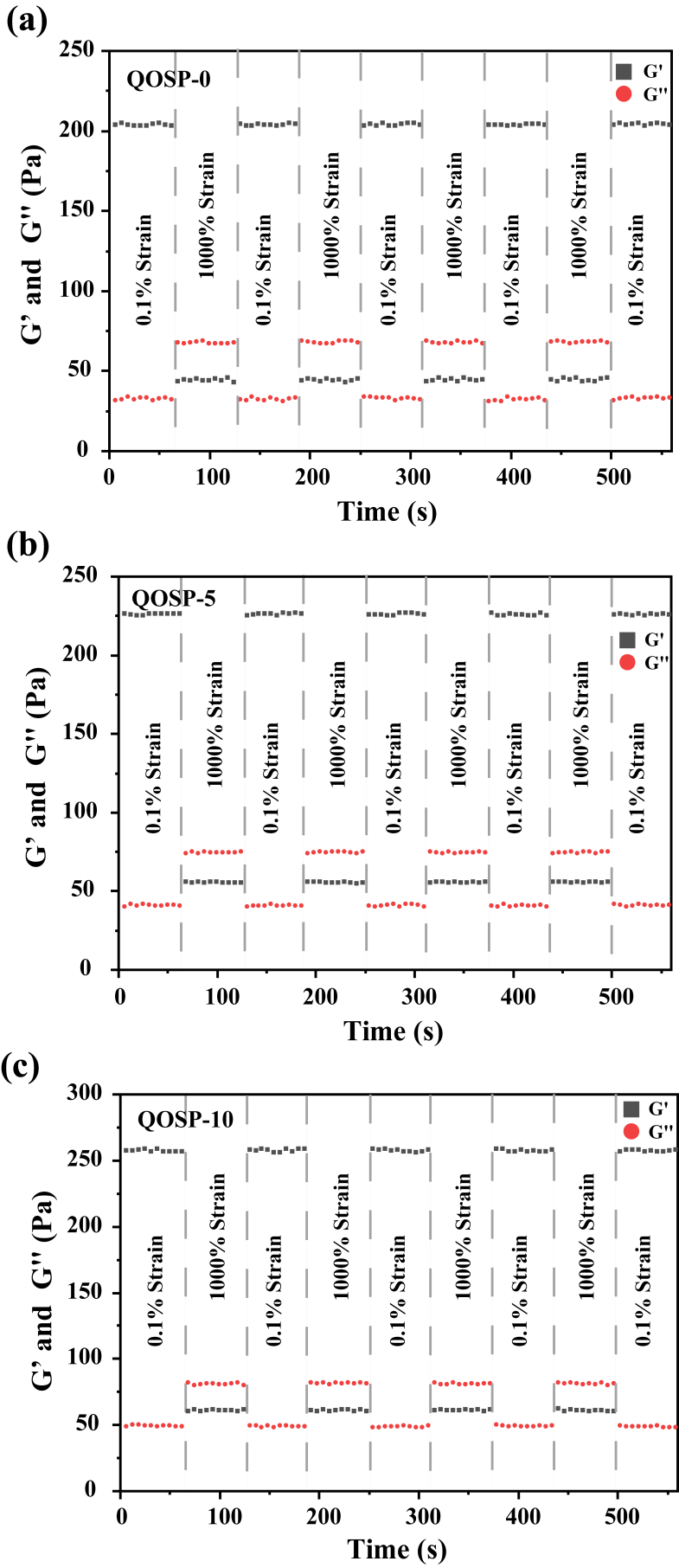


**Figure S7**. Self-healing properties of QOSP-0 (a), QOSP-5 (b) and QOSP-10 (c) hydrogels.


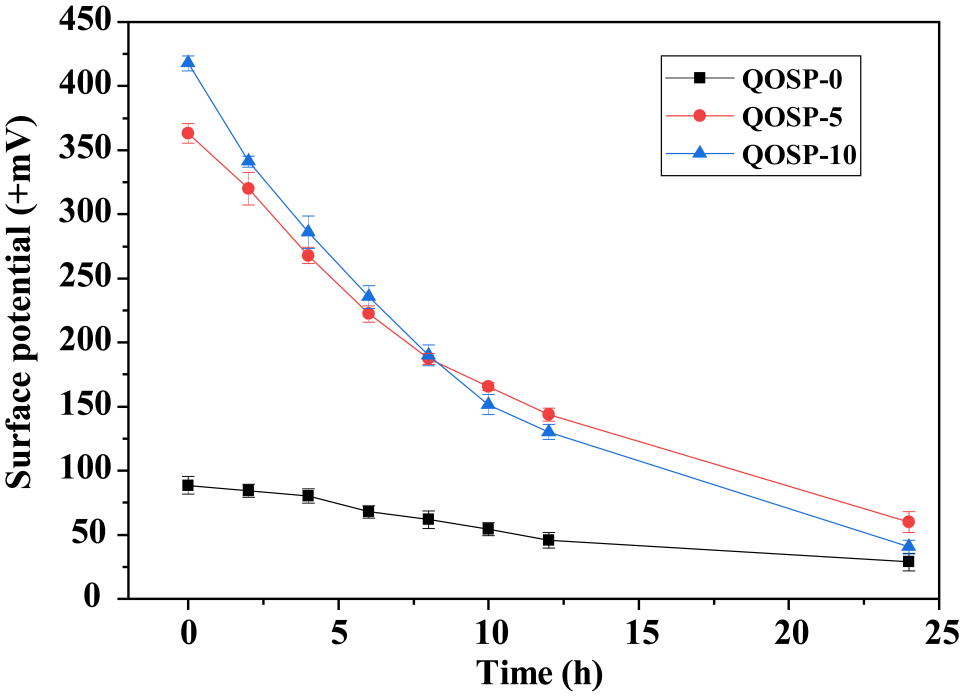


**Figure S8**. Surface potential attenuation curve of QOSP-0, QOSP-5 and QOSP-10 hydrogels.


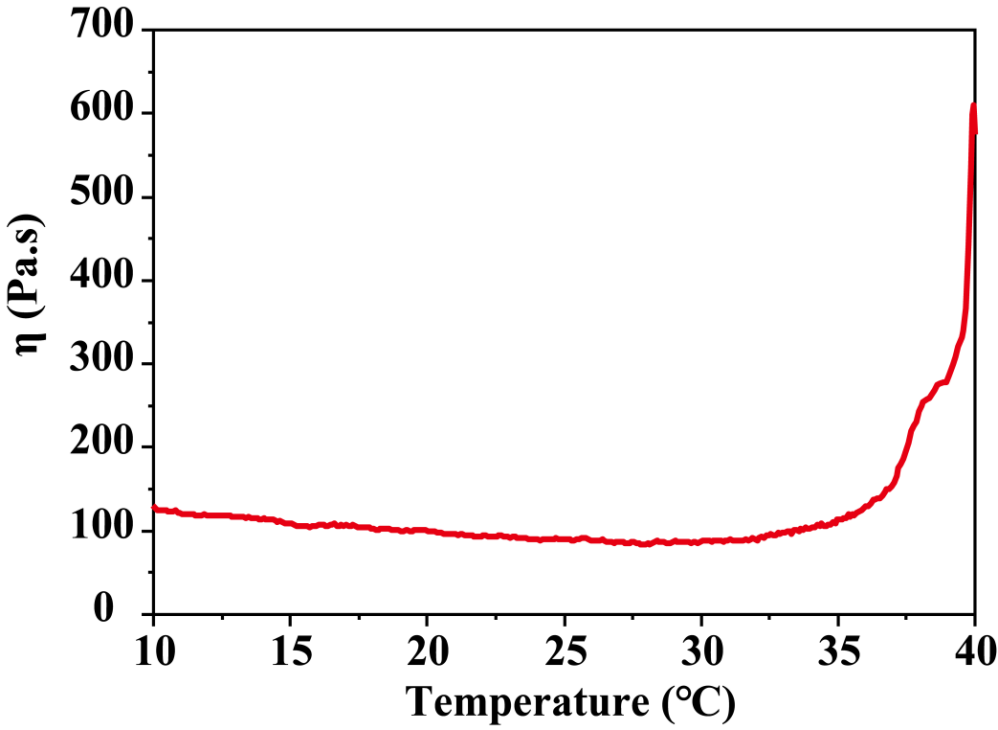


**Figure S9**. Rheological curve of QOSP-5 hydrogel viscosity versus temperature.


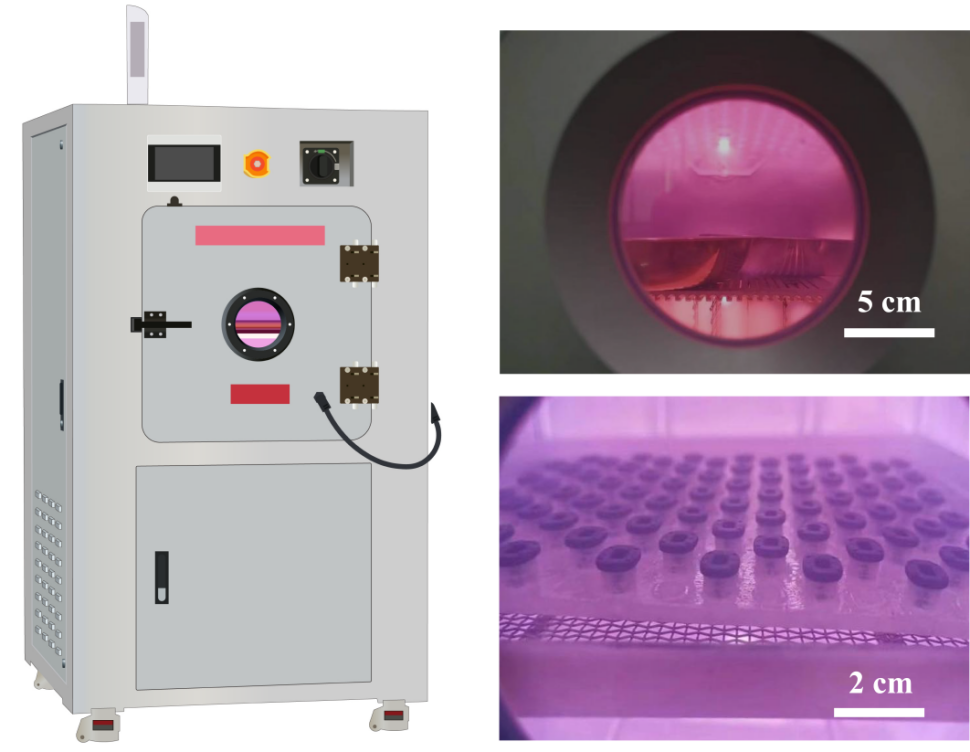


**Figure S10**. Charge injection process in coupled plasma etching.


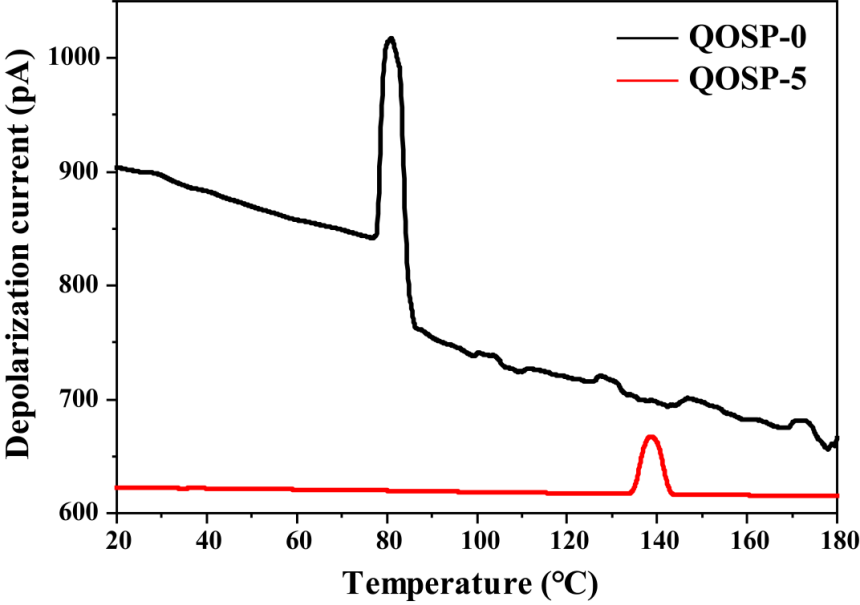


**Figure S11**. TSDC curves of QOSP-0 and QOSP-5 hydrogels.

**Table S1**. Summary of conductive hydrogels composed of different materials and their applications in biology.

| **Composition** | **Power supply principles** | **Conductivity [S/m]** | **Application** | **Ref.** |
| --- | --- | --- | --- | --- |
| chitosan/gelatin/BP@PDA nanosheets | capacitive coupling | 0.289 | Nerve Regeneration | ^[10]^ |
| Alg-PBA/ PVA/Hydroxylated graphene (GOH) | External power supply | 2.26 × 10−3 | Infected joint wounds treatment and detection | ^[11]^ |
| GelMA/alginate/polypyrrole/graphene | External power supply | 6.15 to 12.4 | Cardiac tissue engineering | ^[12]^ |
| PAM/TOCNs/BP@PDA nanosheets | External power supply | 0.41 ± 0.02 | multifunctional wearable sensors | ^[13]^ |
| Lysine/PAAm | External power supply | 73.6 | Wearable strain sensors | ^[14]^ |
| Hydroxypropylcellulose/PVA | External power supply | 3.4 | Artificial tissue | ^[15]^ |
| PEDOT:PSS | External power supply | 4 × 104 | Sensor | ^[16]^ |
| TA/SF/rGO | External power supply | 1 | Bionics, soft robotics | ^[17]^ |
| Polyacrylamide (PAAm)/carbon nanotubes (CNTs) | External power supply | 0.3 | Wearable electronics, strain sensors | ^[18]^ |
| Gelatin/PANI | External power supply | 0.2 | Flexible electronics, biosensors | ^[19]^ |
| PEDOT/agarose | External power supply | 0.1-0.4 | Neural interfaces, bioelectronics | ^[20]^ |
| Alginate/silver nanowires | External power supply | 0.8 | Tissue engineering, biosensors | ^[21]^ |
| PAAm/MXene | External power supply | 1.5 | Flexible electronics, sensors | ^[22]^ |
| Chitosan/polypyrrole (PPy) | External power supply | 0.7 | Motion sensors, medical monitoring | ^[23]^ |
| Polyvinyl alcohol (PVA)/GO | Self-powered (piezoelectric generator) | 0.3 | Wearable sensors, bioenergy harvesting | ^[24]^ |
| Alginate/PANI | External power supply | 0.2 | Bioelectronics, sustainable devices | ^[25]^ |
| Polyacrylic acid (PAA)/carbon nanotubes (CNTs) | External power supply | 1.3 | Wearable electronics, strain sensors | ^[26]^ |


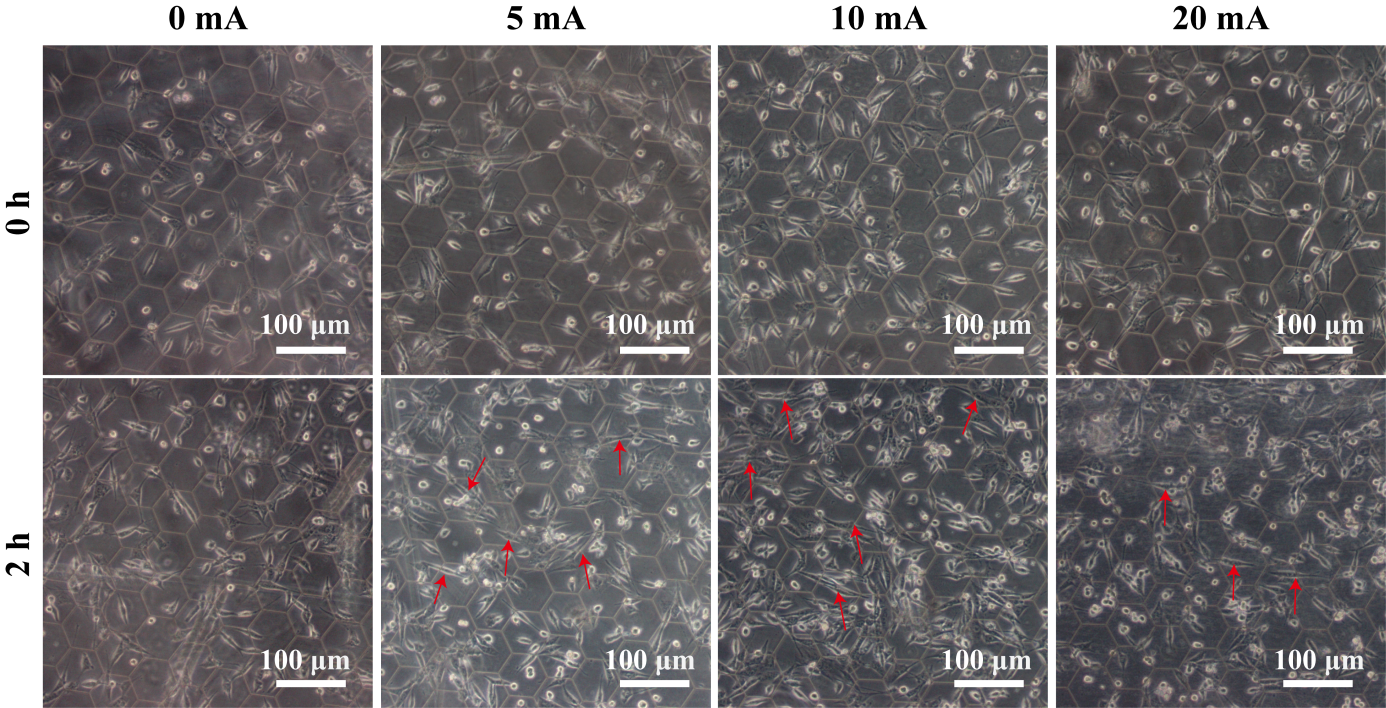


**Figure S12.** Changes in morphology and tropism of NIH/3T3 cells after 2h of electrical stimulation with different current intensities (0 mA, 5 mA, 10 mA, and 20 mA). Scale bar: 100 μm.


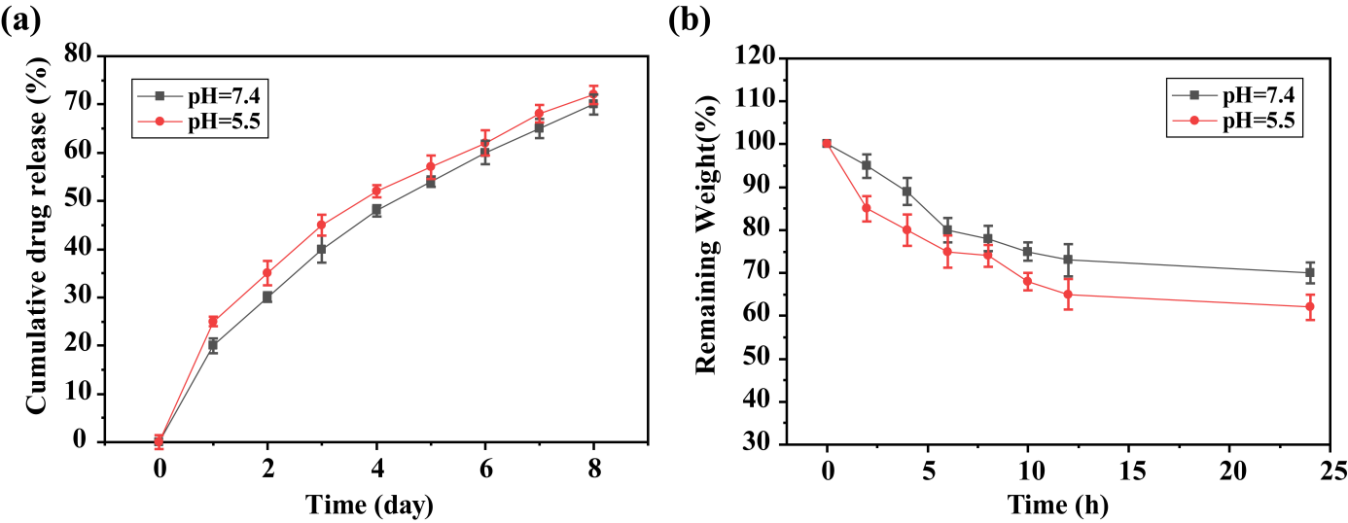


**Figure S13.** (a) Release profiles of the QOSP-5 hydrogels for SDI at pH = 7.4 and pH = 5.5. (b) Degradation profiles of QOSP-5 hydrogels in PBS with pH = 7.4 and pH = 5.5 at 37 °C.


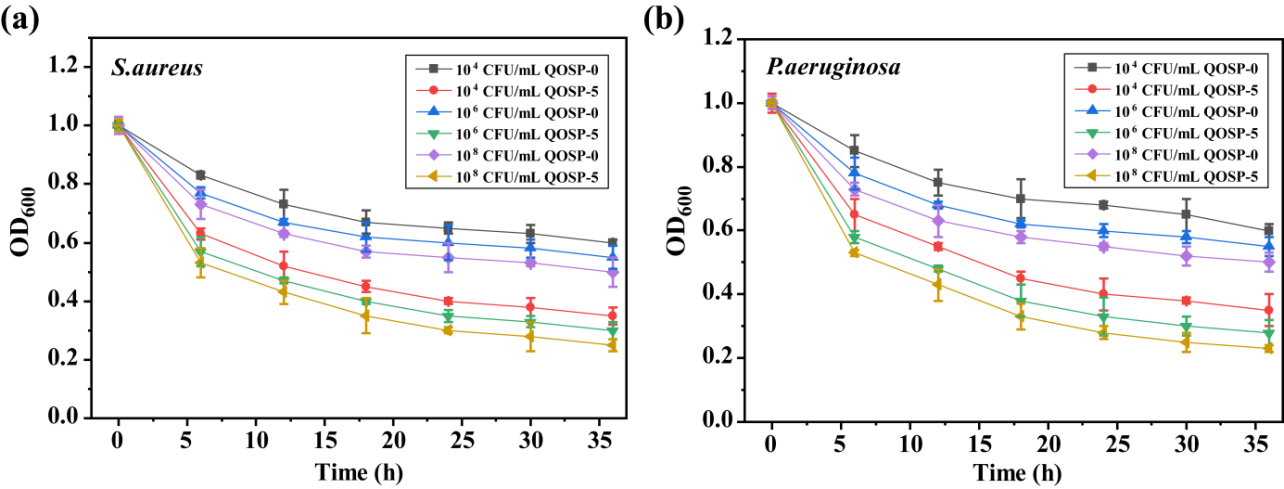


**Figure S14.** Growth curves for SA (a) and PA (b) at various concentrations after QOSP-5 hydrogel treatment.


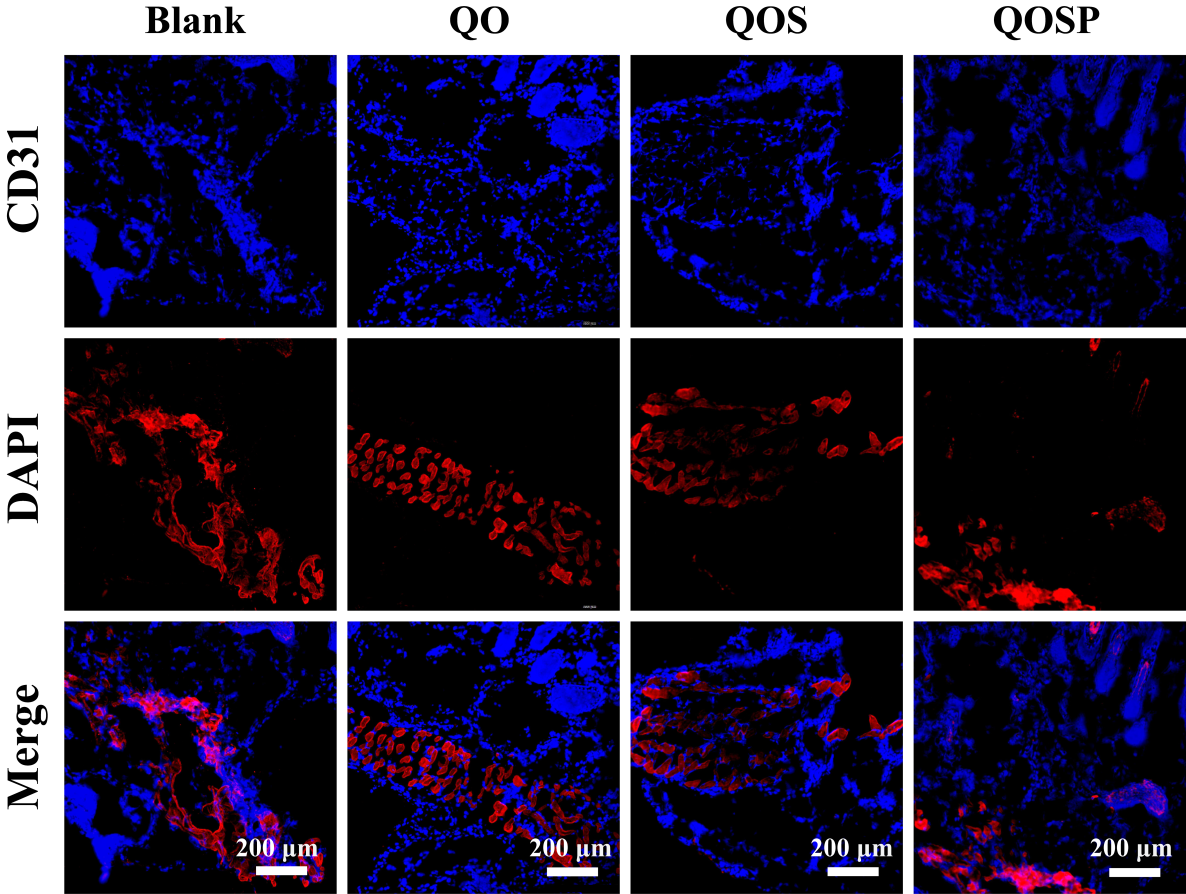


**Figure S15.** Images of immunofluorescence labeling of the regenerated tissue of wounds stained with CD31 on day 10. Scale bar: 200 μm.


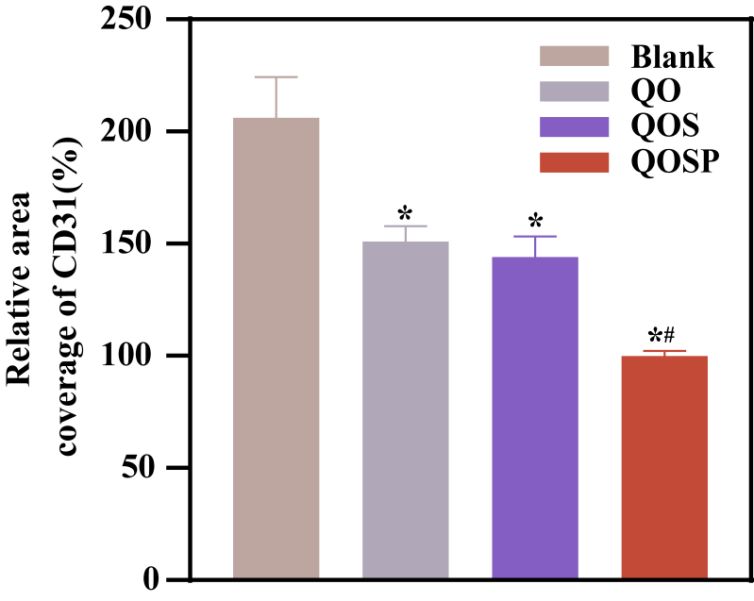


**Figure S16.** Quantitative analysis of relative area percentage of CD31. Data were shown as mean ± SD (n=3,* *p*<0.05 when compared with Blank group; # *p*<0.05 when compared with QO group).


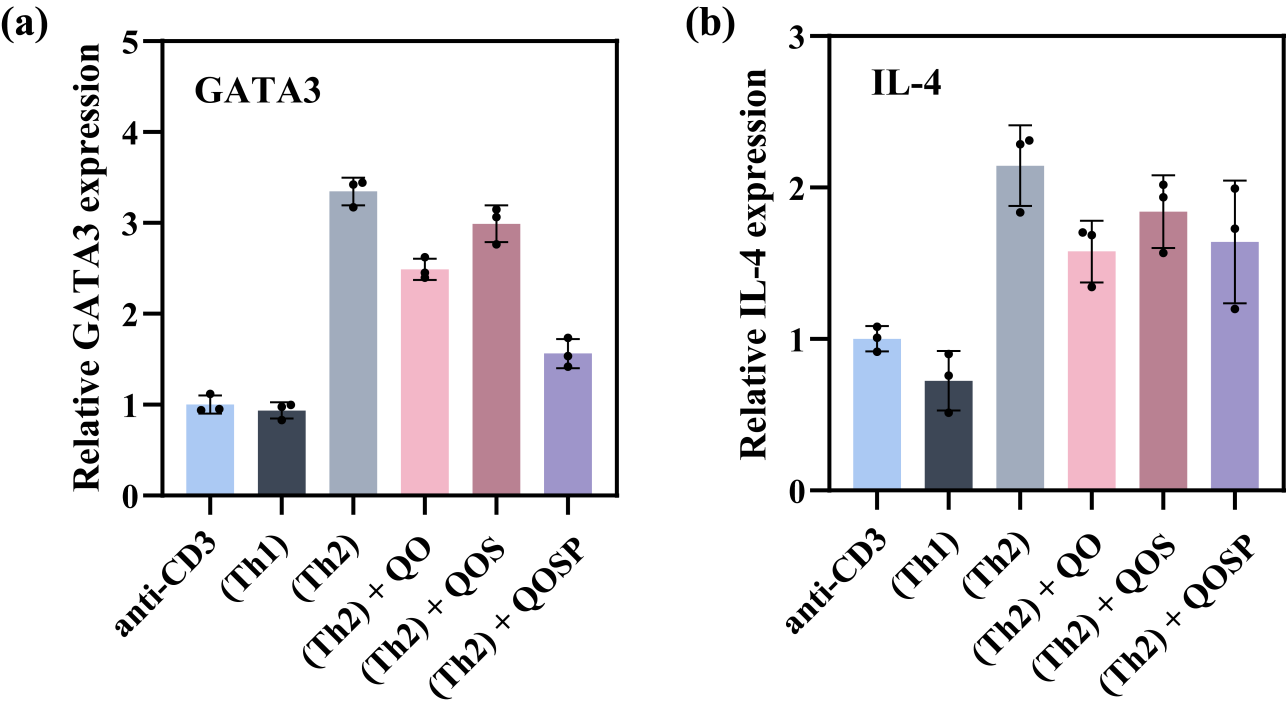


**Figure S17.** (a)-(b) The relative mRNA expression levels of GATA3 and IL-4, specific markers of Th2 cells, indicating the expression level of Th2 cells. In the experiment, Jurkat cells activated with anti-CD3 were directed towards Th1 and Th2 differentiation, respectively, where *(Th1) represents the induction of Th1 environment with anti-CD3/IL-2/IL-12/anti-IL-4, and *(Th2) represents the induction of Th2 environment with anti-CD3/IL-2/IL-4/anti-IFN-γ. The cells were then treated with QO, QOS, and QOSP hydrogels for 24 hours, and the mRNA expression levels were measured.


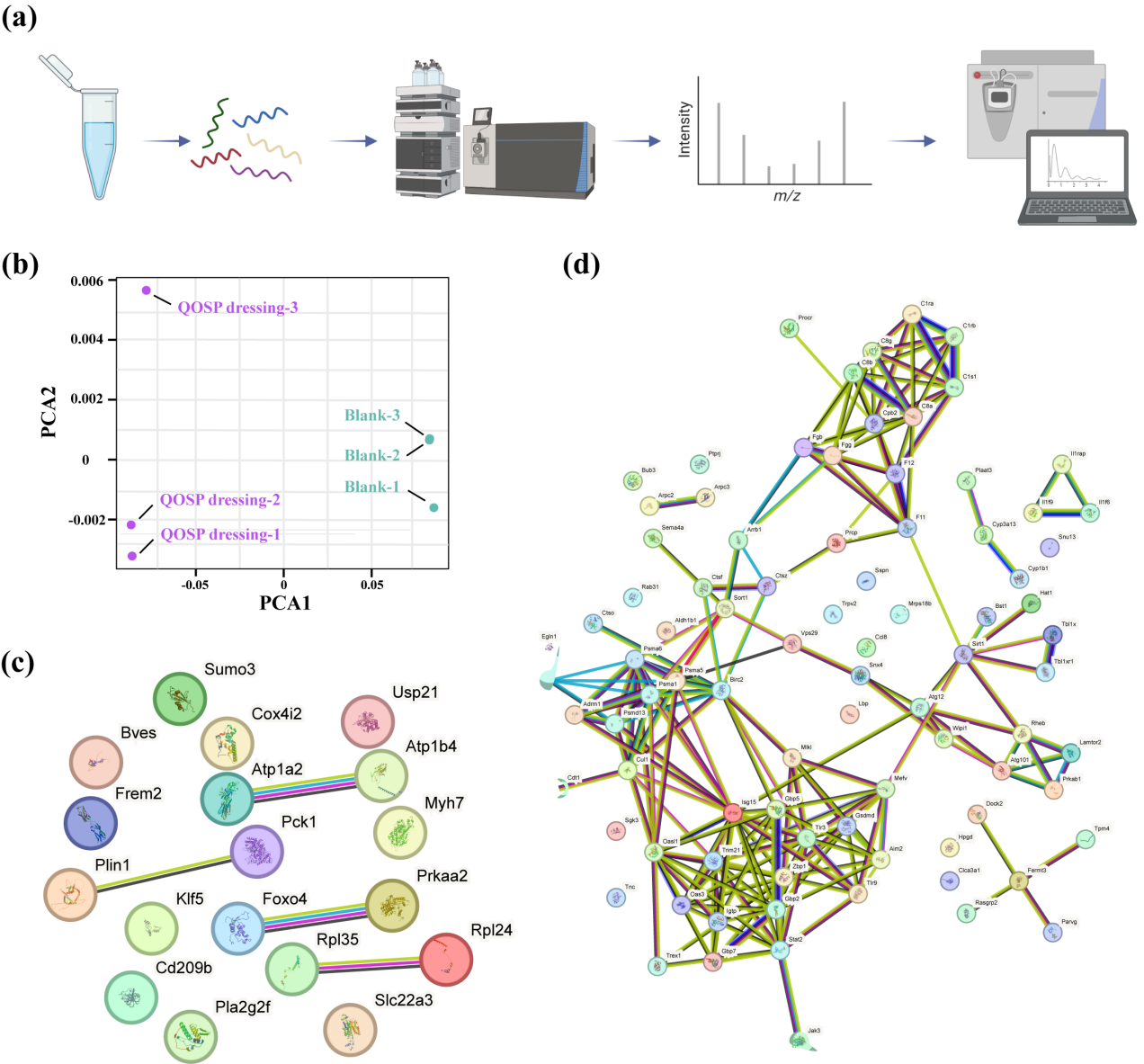


**Figure S18.** (a) A schematic depiction of tissue collection and proteomics analysis. (b) PCA results. (c) PPI network analysis of up-regulated representative DEPs. (d) PPI network analysis of down-regulated representative DEPs.

**Table S2**. Detailed identification of proteins significantly down-regulated in KEGG and Reactome pathways.

| **No.** | **Protein Group** | **Protein Names** | **Genes** | **FC** | ***P*-value** | **First.Protein.Description** |
| --- | --- | --- | --- | --- | --- | --- |
| 1 | Q8CFG9 | C1RB | C1rb | -3.60 | 1.727E-02 | Complement C1r-B subcomponent |
| 2 | Q8CG14 | CS1A | C1s1 | -1.40 | 6.725E-03 | Complement C1s-1 subcomponent |
| 3 | Q8CG16 | C1RA | C1ra | -1.45 | 1.383E-04 | Complement C1r-A subcomponent |
| 4 | Q8CHQ0 | FBX4 | Fbxo4 | -1.07 | 1.498E-02 | F-box only protein 4 |
| 5 | Q8K0E8 | FIBB | Fgb | -1.85 | 1.259E-03 | Fibrinogen beta chain |
| 6 | Q8K182 | CO8A | C8a | -1.58 | 3.206E-05 | Complement component C8 alpha chain |
| 7 | Q8K1B8 | URP2 | Fermt3 | -1.03 | 2.460E-03 | Fermitin family homolog 3 |
| 8 | Q8R2P1 | KLH25 | Klhl25 | -1.76 | 3.655E-02 | Kelch-like protein 25 |
| 9 | Q8R2S8 | CD177 | Cd177 | -2.06 | 1.059E-04 | CD177 antigen |
| 10 | Q8R3E3 | WIPI1 | Wipi1 | -5.18 | 1.603E-03 | WD repeat domain phosphoinositide-interacting protein 1 |
| 11 | Q8VCG4 | CO8G | C8g | -1.60 | 2.436E-03 | Complement component C8 gamma chain |
| 12 | Q8VCM7 | FIBG | Fgg | -1.70 | 1.929E-04 | Fibrinogen gamma chain |
| 13 | Q91X72 | HEMO | Hpx | -1.60 | 2.236E-06 | Hemopexin |
| 14 | Q91Y47 | FA11 | F11 | -1.23 | 3.465E-04 | Coagulation factor XI |
| 15 | Q99MB1 | TLR3 | Tlr3 | -1.38 | 6.099E-03 | Toll-like receptor 3 |
| 16 | Q9CQY1 | ATG12 | Atg12 | -4.54 | 2.849E-03 | Ubiquitin-like protein ATG12 |
| 17 | Q9CZV8 | FXL20 | Fbxl20 | -1.92 | 3.330E-02 | F-box/LRR-repeat protein 20 |
| 18 | Q9D2Y4 | MLKL | Mlkl | -1.40 | 1.058E-03 | Mixed lineage kinase domain-like protein |
| 19 | Q9D8Z6 | ATGA1 | Atg101 | -2.46 | 2.095E-02 | Autophagy-related protein 101 |
| 20 | Q9EQH2 | ERAP1 | Erap1 | -1.50 | 1.891E-03 | Endoplasmic reticulum aminopeptidase 1 |
| 21 | Q9ESY9 | GILT | Ifi30 | -1.06 | 4.229E-02 | Gamma-interferon-inducible lysosomal thiol reductase |
| 22 | Q9JHH6 | CBPB2 | Cpb2 | -1.17 | 1.142E-02 | Carboxypeptidase B2 |
| 23 | Q9JKV1 | ADRM1 | Adrm1 | -1.14 | 8.192E-04 | Proteasomal ubiquitin receptor ADRM1 |
| 24 | Q9QUG9 | GRP2 | Rasgrp2 | -1.99 | 1.019E-02 | RAS guanyl-releasing protein 2 |
| 25 | Q9QUM9 | PSA6 | Psma6 | -1.75 | 3.859E-05 | Proteasome subunit alpha type-6 |
| 26 | Q9QY24 | ZBP1 | Zbp1 | -1.73 | 2.588E-04 | Z-DNA-binding protein 1 |
| 27 | Q9QZU9 | UB2L6 | Ube2l6 | -1.56 | 3.461E-03 | Ubiquitin/ISG15-conjugating enzyme E2 L6 |
| 28 | Q9R013 | CATF | Ctsf | -1.50 | 1.046E-02 | Cathepsin F |
| 29 | Q9R1P4 | PSA1 | Psma1 | -1.23 | 2.004E-03 | Proteasome subunit alpha type-1 |
| 30 | Q9WTX6 | CUL1 | Cul1 | -1.24 | 6.789E-03 | Cullin-1 |
| 31 | Q9WVA4 | TAGL2 | Tagln2 | -1.05 | 1.410E-03 | Transgelin-2 |
| 32 | Q9WVJ2 | PSD13 | Psmd13 | -1.35 | 1.388E-03 | 26S proteasome non-ATPase regulatory subunit 13 |
| 33 | Q9WVL2 | STAT2 | Stat2 | -1.05 | 1.568E-05 | Signal transducer and activator of transcription 2 |
| 34 | Q9Z2U1 | PSA5 | Psma5 | -1.49 | 5.307E-07 | Proteasome subunit alpha type-5 |
| 35 | Q61790 | LAG3 | Lag3 | -3.88 | 1.751E-02 | Lymphocyte activation gene 3 protein |
| 36 | Q61805 | LBP | Lbp | -2.75 | 1.665E-03 | Lipopolysaccharide-binding protein |
| 37 | Q62137 | JAK3 | Jak3 | -1.73 | 1.143E-02 | Tyrosine-protein kinase JAK3 |
| 38 | Q62192 | CD180 | Cd180 | -1.38 | 5.985E-03 | CD180 antigen |
| 39 | Q62210 | BIRC2 | Birc2 | -1.13 | 2.001E-02 | Baculoviral IAP repeat-containing protein 2 |
| 40 | Q64455 | PTPRJ | Ptprj | -1.05 | 9.151E-04 | Receptor-type tyrosine-protein phosphatase eta |
| 41 | Q64695 | EPCR | Procr | -2.30 | 1.488E-02 | Endothelial protein C receptor |
| 42 | Q6P069 | SORCN | Sri | -1.11 | 7.790E-03 | Sorcin |
| 43 | Q7TMR0 | PCP | Prcp | -1.11 | 7.211E-03 | Lysosomal Pro-X carboxypeptidase |
| 44 | Q7TPD1 | FBX11 | Fbxo11 | -2.73 | 6.738E-05 | F-box only protein 11 |
| 45 | Q80YC5 | FA12 | F12 | -1.72 | 6.484E-04 | Coagulation factor XII |
| 46 | Q8BH35 | CO8B | C8b | -1.60 | 1.234E-04 | Complement component C8 beta chain |
| 47 | Q8BLK9 | KS6C1 | Rps6kc1 | -1.76 | 2.383E-02 | Ribosomal protein S6 kinase delta-1 |
| 48 | Q8BM88 | CATO | Ctso | -3.25 | 2.027E-03 | Cathepsin O |
| 49 | Q8BWG8 | ARRB1 | Arrb1 | -1.33 | 1.218E-02 | Beta-arrestin-1 |
| 50 | Q8C3J5 | DOCK2 | Dock2 | -1.18 | 6.826E-03 | Dedicator of cytokinesis protein 2 |


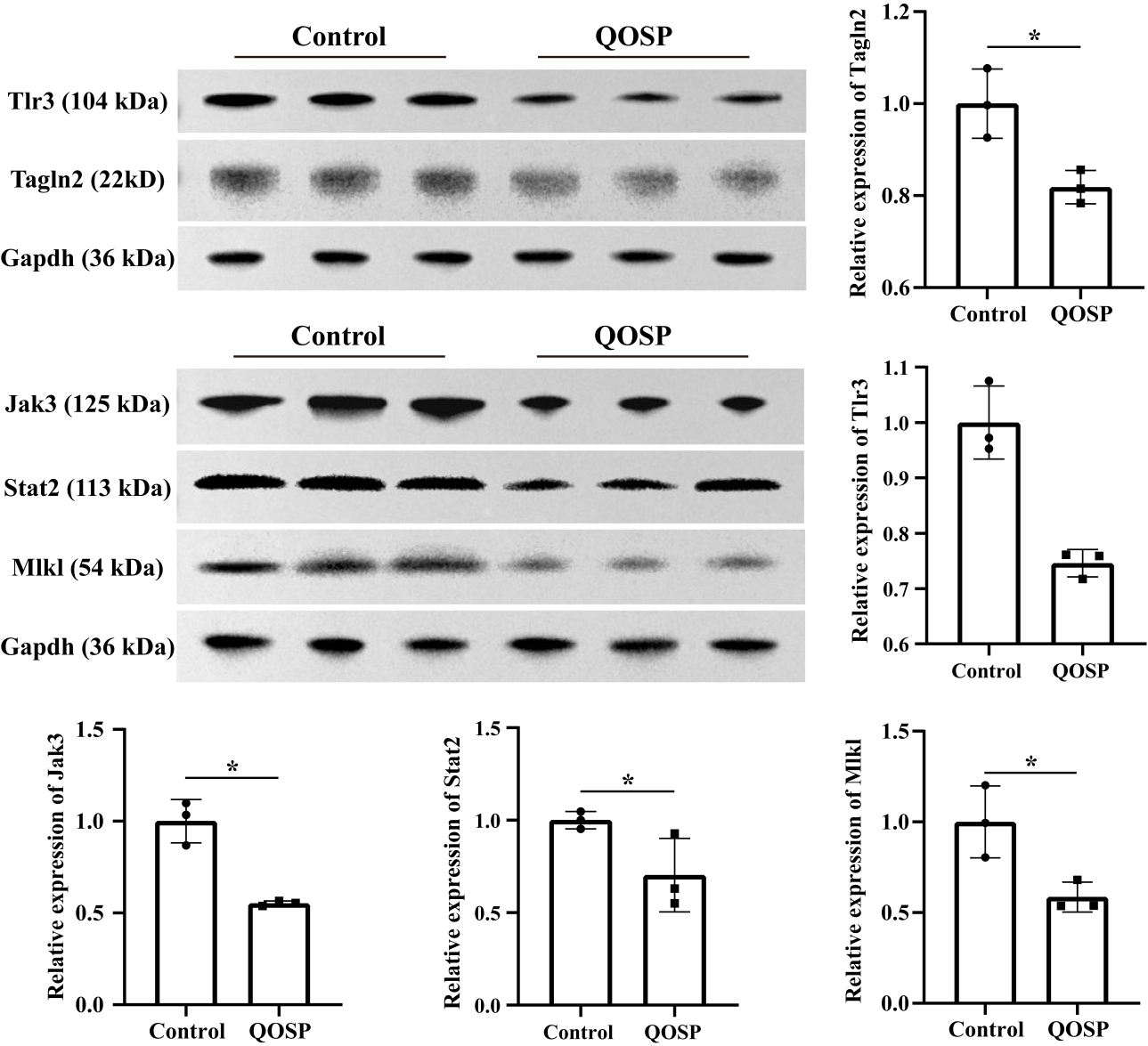


**Figure S19.** Representative western blots for down-regulated proteins. Gapdh was used as the loading control. The quantitative results are expressed as mean ± SD (n = 3), **p* < 0.05.


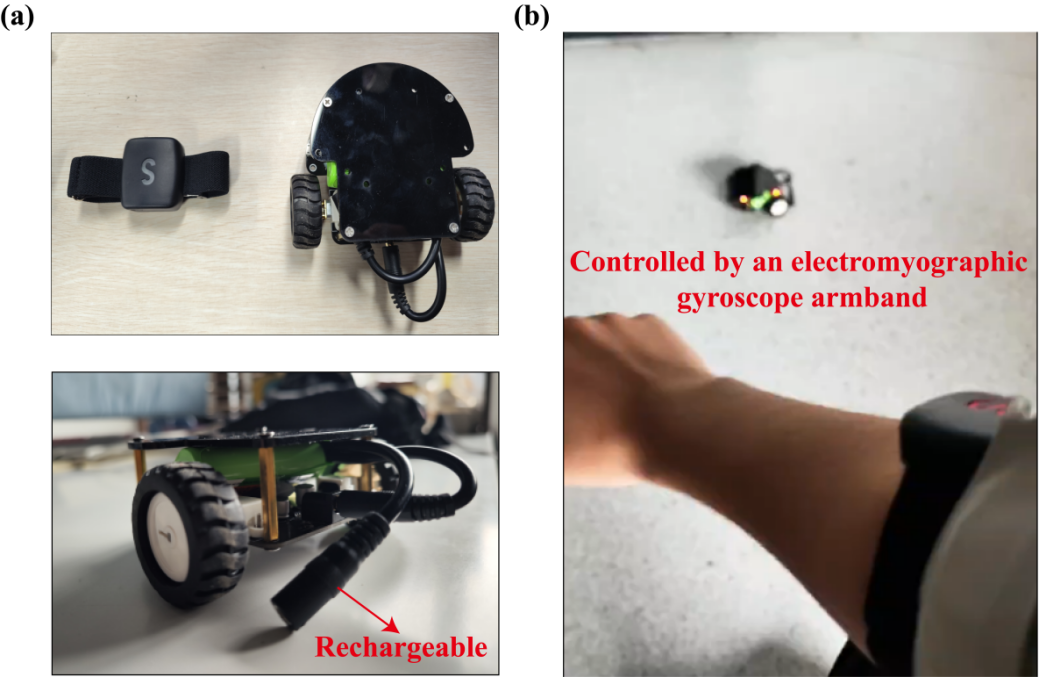


**Figure S20.** (a) Myoelectric gyroscope armband and rechargeable car. (b) Gyroscope-controlled car mechanism: The car can turn according to the armband's gyroscope and change direction by capturing muscle electrical signals.


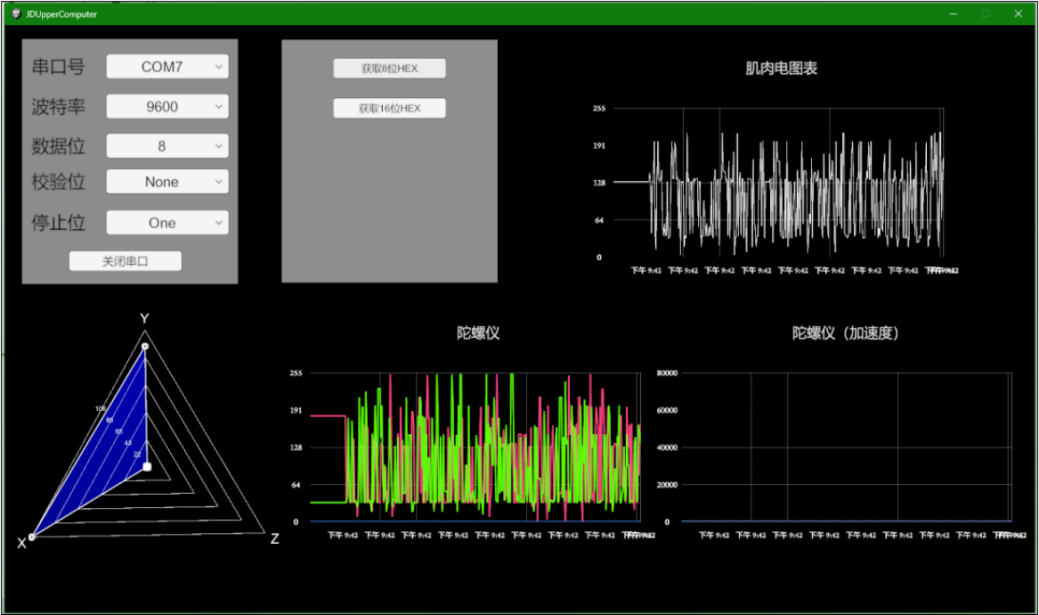


**Figure S21.** EMG gyroscope armband waveform diagram host computer interface.


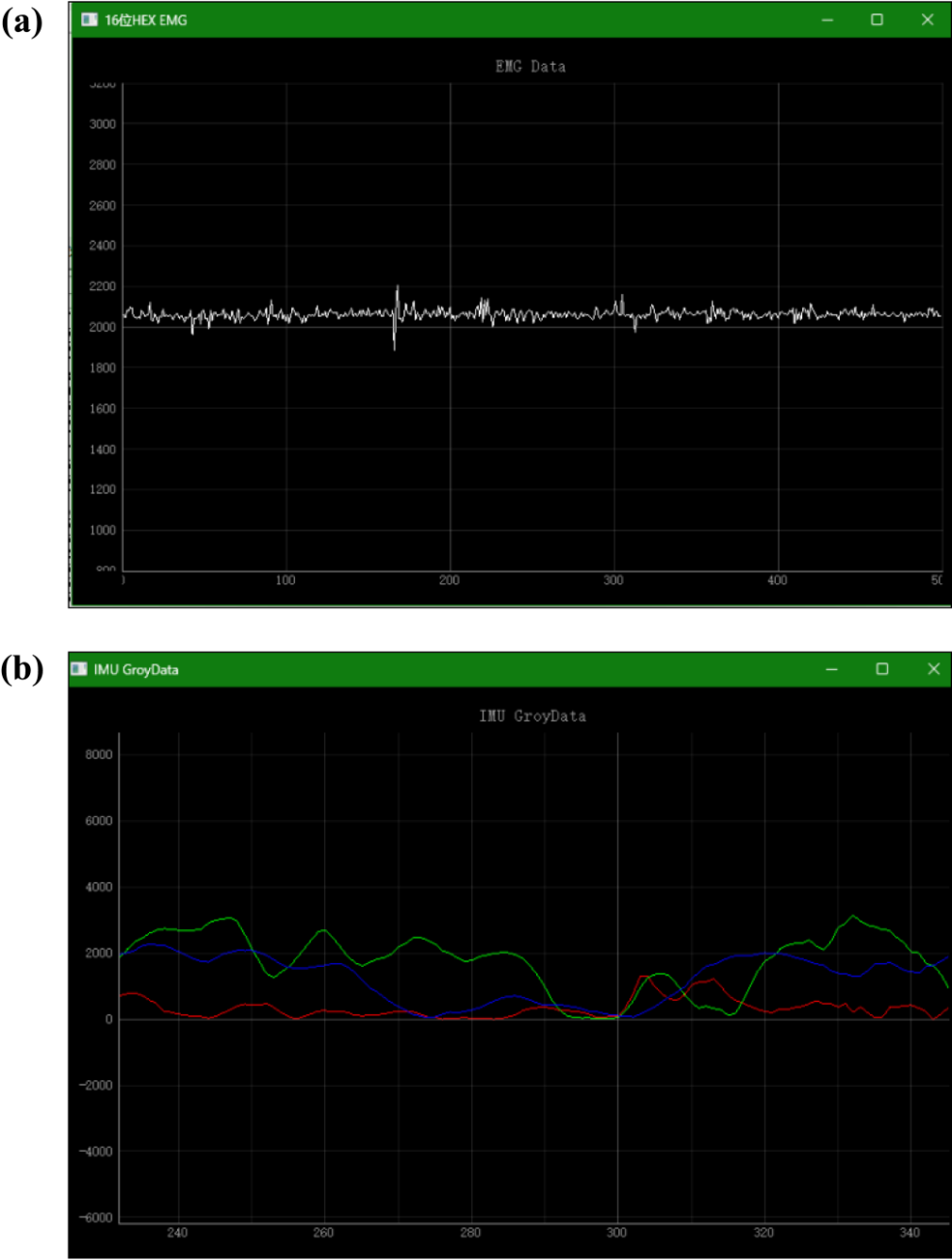


**Figure S22.** Serial port debugging of myoelectric gyroscope armband host computer interface. (a) EMC data. (b) IMU Groydata.

**
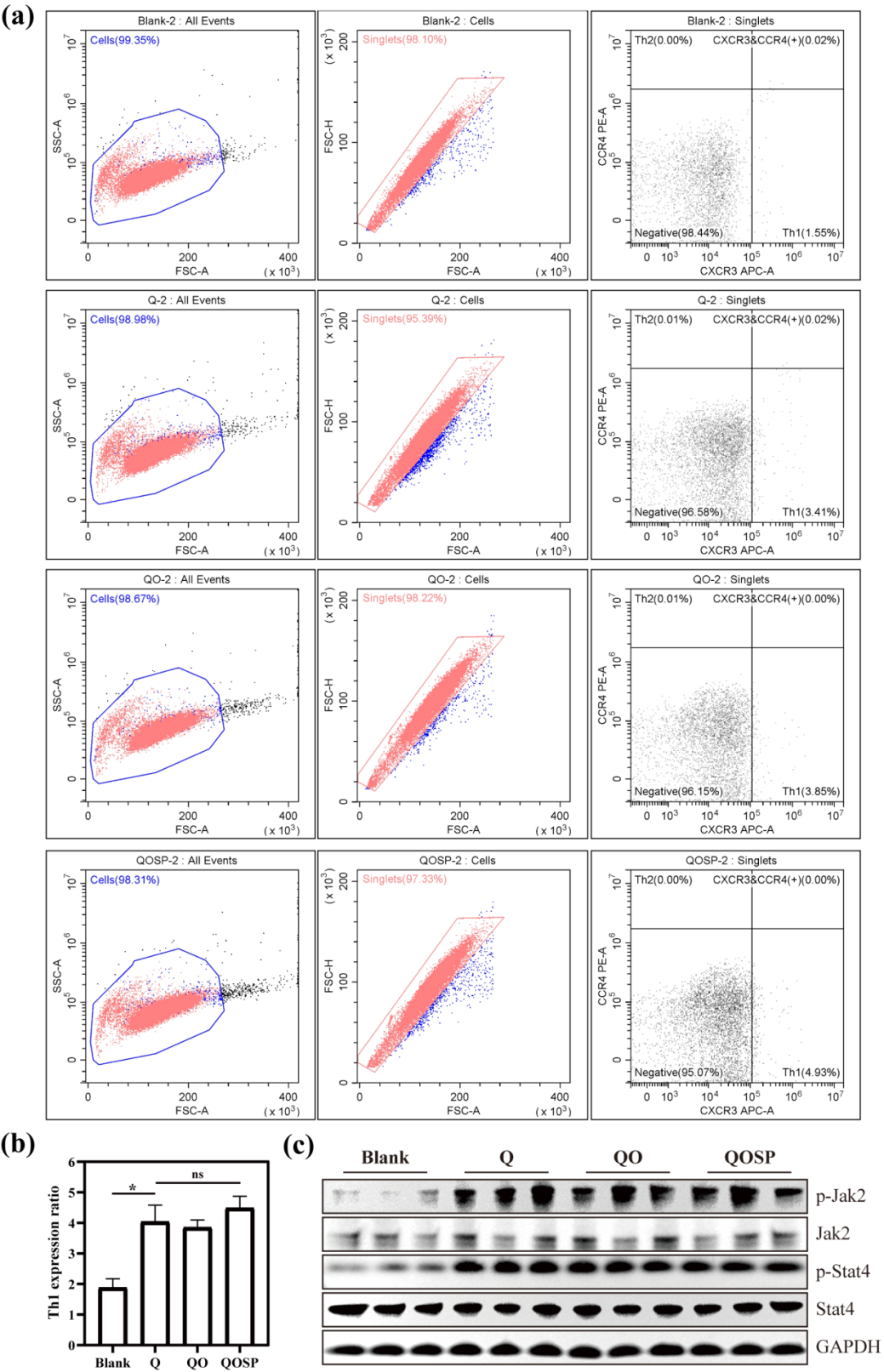
**

**Figure S23.** (a) The expression results of Th1 cells by flow cytometry. (b) The quantitative analysis of the expression ratio of Th1. (c) Western blot analysis of phosphorylated Jak2 and Stat4 proteins. GAPDH was used as the loading control.

**
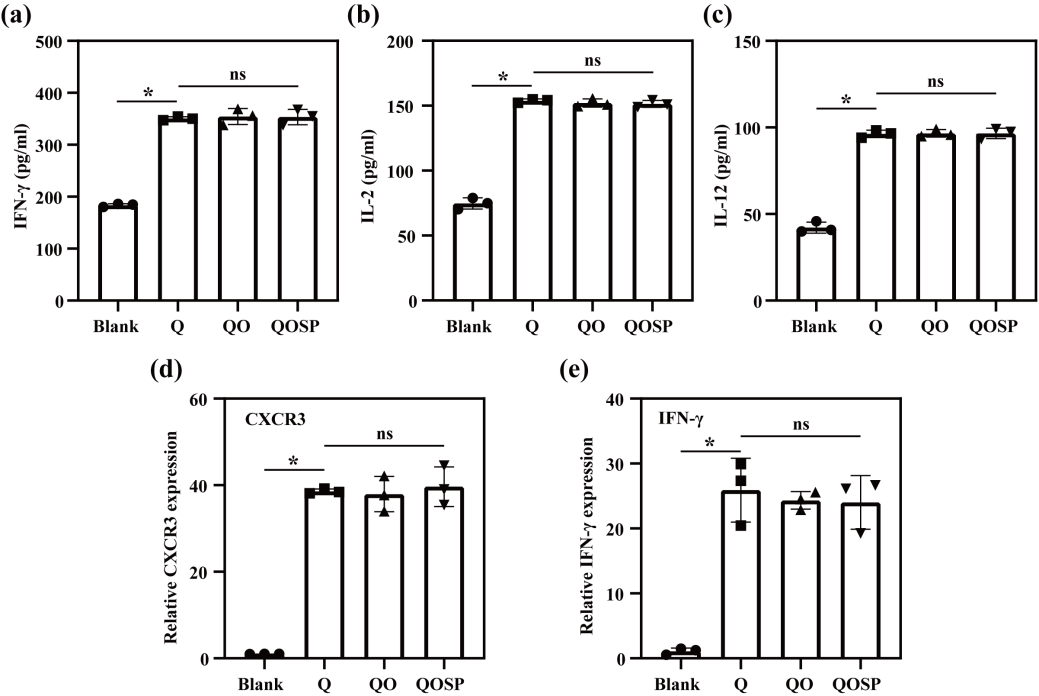
**

**Figure S24.** (a)-(c) Elisa assay results showed the effects of different conditions on the Th1 differentiation of activated Jurkat cells, namely the levels of IFN-γ (a), IL-2 (b), and IL-12 (c). (d)-(e) The relative mRNA expression levels of CXCR3 and IFN-γ, analyzed by real-time qPCR.

# Supplementary References:

[1] J. Qu, X. Zhao, Y. Liang, T. Zhang, P. X. Ma, B. Guo, *Biomaterials* **2018**, *183*, 185.

[2] J. Hu, Y. Quan, Y. Lai, Z. Zheng, Z. Hu, X. Wang, T. Dai, Q. Zhang, Y. Cheng, *J Control Release* **2017**, *247*, 145.

[3] C. Chen, X. Bai, Y. Ding, I.-S. Lee, *Biomater Res* **2019**, *23*, 25.

[4] G. Tai, M. Tai, M. Zhao, *Burns & Trauma* **2018**, *6*, 20.

[5] Z. Liu, X. Wan, Z. L. Wang, L. Li, *Adv Mater* **2021**, *33*, e2007429.

[6] Y. Hu, Y. Wang, F. Yang, D. Liu, G. Lu, S. Li, Z. Wei, X. Shen, Z. Jiang, Y. Zhao, Q. Pang, B. Song, Z. Shi, S. Shafique, K. Zhou, X. Chen, W. Su, J. Jian, K. Tang, T. Liu, Y. Zhu, *Adv Sci* **2024**, *11*, 2307746.

[7] Q.-W. Wang, L.-H. Sun, Y. Zhang, Z. Wang, Z. Zhao, Z.-L. Wang, K.-Y. Wang, G.-Z. Li, J.-B. Xu, C.-Y. Ren, W.-P. Ma, H.-J. Wang, S.-W. Li, Y.-J. Zhu, T. Jiang, Z.-S. Bao, *J Immunother Cancer* **2021**, *9*, e002451.

[8] J. Zhou, F. Wan, L. Wang, C. Peng, R. Huang, F. Peng, *MedComm (2020)* **2023**, *4*, e464.

[9] L. Liu, J.-H. Yen, D. Ganea, *Peptides* **2007**, *28*, 1814.

[10] P. Wu, C. Xu, X. Zou, K. Yang, Y. Xu, X. Li, X. Li, Z. Wang, Z. Luo, *Adv Mater* **2024**, *36*, e2310483.

[11] M. Shan, X. Chen, X. Zhang, S. Zhang, L. Zhang, J. Chen, X. Wang, X. Liu, *Adv Healthc Mater* **2024**, *13*, e2303876.

[12] S. Kaviani, A. Talebi, S. Labbaf, F. Karimzadeh, *Int J Biol Macromol* **2024**, *259*, 129276.

[13] C. Sang, S. Wang, X. Jin, X. Cheng, H. Xiao, Y. Yue, J. Han, *Carbohydr Polym* **2024**, *333*, 121947.

[14] Y. Gao, F. Jia, G. Gao, *Chem Eng J* **2022**, *430*, 132919.

[15] Y. Zhou, C. Wan, Y. Yang, H. Yang, S. Wang, Z. Dai, K. Ji, H. Jiang, X. Chen, Y. Long, *Adv Funct Mater* **2019**, *29*, 1806220.

[16] B. Lu, H. Yuk, S. Lin, N. Jian, K. Qu, J. Xu, X. Zhao, *Nat Commun* **2019**, *10*, 1043.

[17] F. B. Kadumudi, M. Hasany, M. K. Pierchala, M. Jahanshahi, N. Taebnia, M. Mehrali, C. F. Mitu, M.-A. Shahbazi, T.-G. Zsurzsan, A. Knott, T. L. Andresen, A. Dolatshahi-Pirouz, *Adv Mater* **2021**, *33*, e2100047.

[18] Z. Jiang, K. Fukuda, W. Huang, S. Park, R. Nur, Md. O. G. Nayeem, K. Yu, D. Inoue, M. Saito, H. Kimura, T. Yokota, S. Umezu, D. Hashizume, I. Osaka, K. Takimiya, T. Someya, *Adv Funct Mater* **2019**, *29*, 1808378.

[19] G. Wu, L. Wang, K. Song, J. Xu, J. Li, X. Fang, D. Huang, L. Zheng, Q. Wei, W. W. Yu, *Inorg Chem Front.* **2024**.

[20] Z. Ma, P. Li, J. Shi, F. Sun, Y. Fu, Z. Wang, Y. Fang, J. Han, X. Qu, *J Mater Chem A* **2024**.

[21] Q. Fu, C. Zhu, X. Zhao, X. Wang, A. Chaturvedi, C. Zhu, X. Wang, Q. Zeng, J. Zhou, F. Liu, B. K. Tay, H. Zhang, S. J. Pennycook, Z. Liu, *Adv Mater* **2019**, *31*, 1804945.

[22] H. Li, W. Zhang, A. Y. Elezzabi, *Adv Mater* **2020**, *32*, 2003574.

[23] C.-X. Zhao, J.-N. Liu, B.-Q. Li, D. Ren, X. Chen, J. Yu, Q. Zhang, *Adv Funct Mater* **2020**, *30*, 2003619.

[24] W. Cai, P. Zou, S. Rong, H. Wang, X. Chen, Z. Zhang, Y. Wang, C. Liu, T. Yang, T. Niu, S. Jin, W. Tian, J. Yao, S. F. Liu, K. Zhao, *Energ Environ Sci* **2024**.

[25] X. Yang, S. K. Biswas, J. Han, S. Tanpichai, M.-C. Li, C. Chen, S. Zhu, A. K. Das, H. Yano, *Adv Mater* **2021**, *33*, 2002264.

[26] Y. Li, X. Zhang, Y. Zhang, Y. Zhang, Y. He, Y. Liu, H. Ju, *ACS Appl. Mater. Interfaces* **2020**, *12*, 19313.
